# Robots in mine search and rescue operations: a review of platforms and design requirements

**DOI:** 10.3389/frobt.2026.1857704

**Published:** 2026-08-05

**Authors:** R. Bakzadeh, V. Androulakis, H. Khaniani, S. Shao, M. Hassanalian, P. Roghanchi

**Affiliations:** 1 Department of Mining Engineering, University of Kentucky, Lexington, KY, United States; 2 SenseLab Space Informatics, Technical University of Crete, Chania, Crete, Greece; 3 Department of Mineral Engineering, New Mexico Tech, Socorro, NM, United States; 4 Department of Electrical Engineering, Colorado School of Mines, Golden, CO, United States; 5 Department of Mechanical Engineering, New Mexico Tech, Socorro, NM, United States

**Keywords:** coal-mine rescue robots, design requirements, mine emergencies, mine search and rescue operations, robotics

## Abstract

The use of automation in mining can be found at all stages of the mining process, covering exploration, excavation, loading, transportation, mineral processing, and search and rescue missions in emergency situations. Due to the harsh conditions during an underground mine disaster, robots can be of great assistance to rescue teams by entering areas that are unsafe for human rescuers, locating trapped workers, and collecting valuable data. The design and implementation of coal-mine rescue robots are characterized by great complexity because they encompass a great range of components and requirements. However, comprehensive guidelines for the design and deployment of robots in harsh underground environments have not been established. As a result, the developers of coal-mine rescue robots (CMRRs) must exercise their own judgment on the development and evaluation of the platform functionalities. This survey attempts to review design and functionality requirements based on the common practices and lessons learned from the existing CMRRs with the aim of facilitating future research and development on coal-mine search and rescue robots. An updated overview of the documented CMRRs of the last 3 decades showcases the intricate relationship of the common design considerations for CMRR design.

## 1 Introduction

Automation and robotics have become integral to the mining industry and the scientific community, with a vast variety of applications, led by the rapid technological advances in computer architecture and artificial intelligence. Transportation (Hancock et al., 2019; Attoh-Okine and Bullock, 2000) agriculture (Ruckelshausen et al., 2009), naval operations (Sapaty, 2015), medicine and healthcare (Okamura et al., 2010), warehouse and supply chain (Hamberg and Verriet, 2012), construction (Faghihi et al., 2015), industrial production and assembly (Chadeev and Aristova, 2017; Salonitis, 2014), automotive design (Muslim and Itoh, 2019), mining (Paredes and Fleming-Muñoz, 2021), and geotechnical operations (Farook et al., 2019) are some of the industries in which automation has gained a significant role in the last few decades. The drive of the ever-increasing presence of automation can be attributed to the many advantages of automating parts of the working cycles of the different industries or parts of the product. Automation, among other things, allows for speed, accuracy, and efficiency (especially for repetitive tasks), increased safety, higher productivity, reduced operational and maintenance costs, and increased effectiveness of the production line (Gupta and Arora, 2009).

The degree of *autonomy* entrusted to a machine is the basis of the most common classification system for autonomous machines. Sheridan and Verplank (1978) provided one of the first classifications of automation, which consists of ten levels of automation (LoA), ranging from manually operated to fully automated (see Table 1). The appropriate autonomy level to be delegated to a machine is of crucial importance because automation does not simply replace human activity but changes it and imposes new requirements on the involved operators and machines (Parasuraman et al., 2000).

**TABLE 1:** Levels of autonomy classification as proposed by Sheridan and Verplank (1978).

Autonomy level	Description	Details
Level 1	Fully manual control	No computer in the loop
Level 2	Computers offer a complete set of decisions/action options	Several options are provided to humans who decide
Level 3	Computers narrow the selection down to a few options	Humans decide amongst options
Level 4	Computer provides options and suggests one of the options	Humans still decide amongst options
Level 5	Computers execute their own suggestion if the human approves	Human approval needed for execution
Level 6	Computers provide humans with limited time to veto before automatic execution	Limited time for veto given to humans
Level 7	Computers execute automatically and then necessarily inform the human	No human interference, only information monitoring at the end
Level 8	Computers inform humans only if asked	Humans get information only if they ask
Level 9	Computers inform humans only if they decide to	Computers decide whether to give information
Level 10	Fully autonomous control	Computers decide everything and act autonomously, ignoring humans

The increasing deployment of autonomous haulage systems (AHS) alongside their software suites developed by coalitions of mining machinery manufacturers, mining companies, and software development agencies, such as Komatsu’s FrontRunner AHS (Komatsu, 2023). Caterpillar (2023) demonstrates the maturity of automation in routine mining operations. A particular but more complex application is the deployment of robotic agents in search and rescue operations (S&R ops) for mine emergencies (Murphy et al., 2009). While S&R platforms share similarities with disaster robots deployed after earthquakes, floods, and building fires (Gossett, 2020), coal-mine rescue robots (CMRRs) face a specific combination of constraints: potentially explosive atmospheres, GPS-denied navigation, unreliable communications over long distances, dynamic post-disaster terrain, and strict permissibility certification requirements. Robots designed for mine emergency S&R operations, either fully autonomous or in collaboration with humans, could improve the speed, accuracy, and safety of rescue operations in hazardous underground mine environments, and ultimately improve the chances of successful rescue operations. The ability to collect and process vast amounts of data even in environments inaccessible to humans translates directly into reduced exposure of human rescue teams. A robot that precedes human rescuers can map temperature and gas concentrations, locate roof falls and fires, and even assess structural hazards and the condition of trapped, injured, or incapacitated miners. However, rescue robot technology is still far from reliable autonomous operation in these conditions, and human oversight and intervention are required in deployment.

Mine S&R robots share design principles with disaster robots used in earthquakes, building collapses, or other catastrophic events (Li et al., 2020). However, underground coal mines impose a specific combination of constraints not faced by surface or general-purpose disaster robots: potentially explosive atmospheres (due to dissipation of methane from coal seams), unreliable communications, GPS-denied navigation, structurally dynamic post-disaster terrain, and strict permissibility certification. These constraints raise the bar for requirements regarding navigation and localization, communication hardware and protocols, power and energy sufficiency, maintenance factors, hardware enclosures, and human–robot interaction (Li et al., 2020; Dinelli et al., 2023). A coal-mine rescue robot (CMRR) that meets these requirements is, by design, capable of operating in any other underground mine; hence the interchangeable use of the terms CMRR and MRR hereafter.

Several competition programs held in recent years have accelerated research on robotic systems for underground environments. The Defense Advanced Research Projects Agency’s (DARPA) Subterranean Challenge targeted autonomous systems that can map and navigate complex underground environments, such as tunnel systems, underground networks in urban areas, and cave systems (DARPA, 2022). The RoboCupRescue aims to boost awareness and development of robotic platforms capable of being the main actors in S&R ops (RoboCup Federation, 2023). NASA’s Space Robotics Challenge focuses on underground environments, as well as simulated caves, lava tubes, and space exploration (NASA, 2019). euRobotics organizes the European Robotics League tournaments featuring challenges related to urban search and rescue scenarios that can also take place underground (euRobotics, 2022). These programs have produced valuable advances in autonomous navigation, mapping, and multi-agent coordination, but none has targeted (coal-)mine-specific deployment conditions.

Some existing works that discuss mine rescue robots either predate the latest technological advances, address a narrow technological or geographic subset of platforms, or are not primarily review articles but technical studies whose literature sections touch on prior CMRRs (Table 2). Murphy et al. (2009), which is the closest precedent to a dedicated review, covers four operationally deployed MRRs with a mission- and failure-focused perspective, but it predates more than 15 years of subsequent platform development. Green (2013) is a requirement-focused overview rather than a systematic platform review. Reddy et al. (2015) provide a brief general overview of five platforms without detailed failure or deployment analysis. Zhao et al. (2017), Wang et al. (2018), and Novák et al. (2018) are primarily technical articles on specific platforms (CUMT/MSRBOTS, MINBOT, and TeleRescuer/MPI, respectively) whose related-work sections discuss some earlier CMRRs. Wang et al. (2018) and Li et al. (2020) are the strongest sources for development barriers and application history, covering five and eight platforms, respectively, but neither provides a unified design-requirement taxonomy. Zhai et al. (2020) cover ten platforms but exclusively through the perspective of binocular vision systems, and Li et al. (2022) offer a lessons-learned perspective on eight platforms without structured design-requirement coverage. Bołoz and Biały (2020) provide a broad underground mining automation review that mentions seven CMRR-relevant platforms. None of these works cover all 15 documented CMRRs.

**TABLE 2:** Overview of existing works discussing mine rescue robots and their coverage of the 15 CMRR platforms identified in the present survey.

Source	Year	# Of CMRRs	CMRRs identified	Comment
Murphy et al.	2009	4/15	Numbat ANDROS Wolverine/V2 Groundhog Inuktun VGTV	Mission/failure focused. Overview of real robot-assisted mine rescue deployments. Discusses mission/failure details and operational limitations
Green	2013	6/15	RATLER Numbat ANDROS Wolverine/V2 Groundhog Cave Crawler Gemini-Scout	Requirement-focused review. Not a broad platform review
Reddy et al.	2015	5/15	ANDROS Wolverine/V2 Gemini-Scout Numbat Groundhog Subterranean Robot	General and brief review of MRRs
Zhao et al.	2017	5/15	ANDROS Wolverine/V2 Groundhog; Gemini-Scout MINBOT-II Mobile Inspection Platform (MPI)	Primarily a technical article on MSRBOTS/CUMT-type systems. Literature review section briefly discusses earlier CMRRs
Wang et al.	2018	5/15	RATLER ANDROS Wolverine/V2 Western Australia Water Service Company Robot Leader CUMT-V	Partly mission/deployment focused. Discusses CMRR development barriers (e.g., certification, market demand, and technical integration) and practical deployment problems
Novák et al.	2018	6/15	MINBOT-II Numbat ANDROS Wolverine/V2 Gemini-Scout Mobile inspection Platform (MPI) TeleRescuer	Literature/background section focuses on explosion safety regulations and design requirements for mobile robots in coal mines. Discusses permissibility/explosion-proof design
Li et al.	2020	8/15	RATLER ANDROS Wolverine/V2 MINBOT-II Groundhog Numbat Souryu series/Souryu-V TeleRescuer CUMT-V	Mission/application focused. Discusses multiple CMRRs and reasons for the unsuccessful deployment of CMRRs
Zhai et al.	2020	10/15	RATLER ANDROS Wolverine/V2 Groundhog Cave Crawler Numbat WA Water Service Company Robot Leader MINBOT-II CUMT-V TeleRescuer	Broad review of CMRRs focused on binocular vision technology. Mission/failure discussion is brief due to perception focus
Bołoz and Biały	2020	7/15	Groundhog ANDROS Wolverine/V2 Gemini-Scout Numbat WA Water Service Company Mobile Inspection Platform/MPI TeleRescuer	Broad underground mining automation review
Wang et al.	2014	4/15	ANDROS Wolverine/V2 Groundhog Gemini-Scout MINBOT-II	Primarily a technical article on MINBOT-I/II. Literature review section briefly discusses earlier CMRRs
Li et al.	2022	8/15	RATLER ANDROS Wolverine/V2 Numbat Souryu series/Souryu-V Groundhog WA Water Service Company Robot CUMT-V TeleRescuer	Mission/application and lessons-learned focused. Discusses CMRR development history, applications, and lessons learned. Discusses broader design requirements and practical deployment barriers

The present survey therefore focuses on a systematic description of the mechanical design and sensor suite combinations of 15 documented CMRRs developed over the last 3 decades, together with a structured discussion of the design requirements that govern CMRR development. The aim is to comprehensively present CMRR platform design, sensing architecture, autonomy level, communication approach, explosion-proof and waterproof certification, deployment status, and design trade-offs within a single unified review.

Three areas are deliberately not within scope. First, the autonomy, mapping, SLAM, path planning, and software architecture of CMRRs are not reviewed: the field is far too vast to treat alongside hardware in a single review, and the source literature for most of the reviewed platforms provides little information on these topics because development emphasis has been on hardware and certification. Second, communication systems are reviewed only at the level of physical channel (wired/wireless, fiber-optic, mesh) and bandwidth requirement; the protocol stack and signal-level performance under coal-mine radiofrequency (RF) conditions are research topics in their own right and are not treated here. Third, an aggregated statistical analysis of CMRR failure modes is not feasible: only two sources, namely Murphy et al. (2009) and Li et al. (2022), provide substantive lessons learned and failure analysis from real deployments. The remaining works offer insufficient mission-level detail to support a cumulative failure analysis. Moreover, only one platform (see Section 3.3) has a multi-deployment operational record, and the available information on the type and root cause of failures is fragmentary. Where the source literature does report a specific failure, it is described in Section 3 for the relevant platform.

This study reviews the documented CMRRs of the last 3 decades and identifies the principal design requirements that govern their mechanical and computational architecture. The aim is to support future development of robotic systems that help mine rescue responders complete their mission faster and more safely. Successful mine rescue deployment depends on a broader ecosystem, including surface and subsurface detection systems, command-and-control frameworks, multi-agent coordination, and rescue tactics, that lie outside this review’s scope. Recent advances in multi-agent systems (e.g., DARPA Subterranean Challenge) and self-supervised robotics offer promising paths for those systemic challenges and warrant separate treatment. The present work focuses on the mechanical and computational design of individual platforms, the trade-offs among them, and the lessons learned from documented field deployments.

**FIGURE 1:**
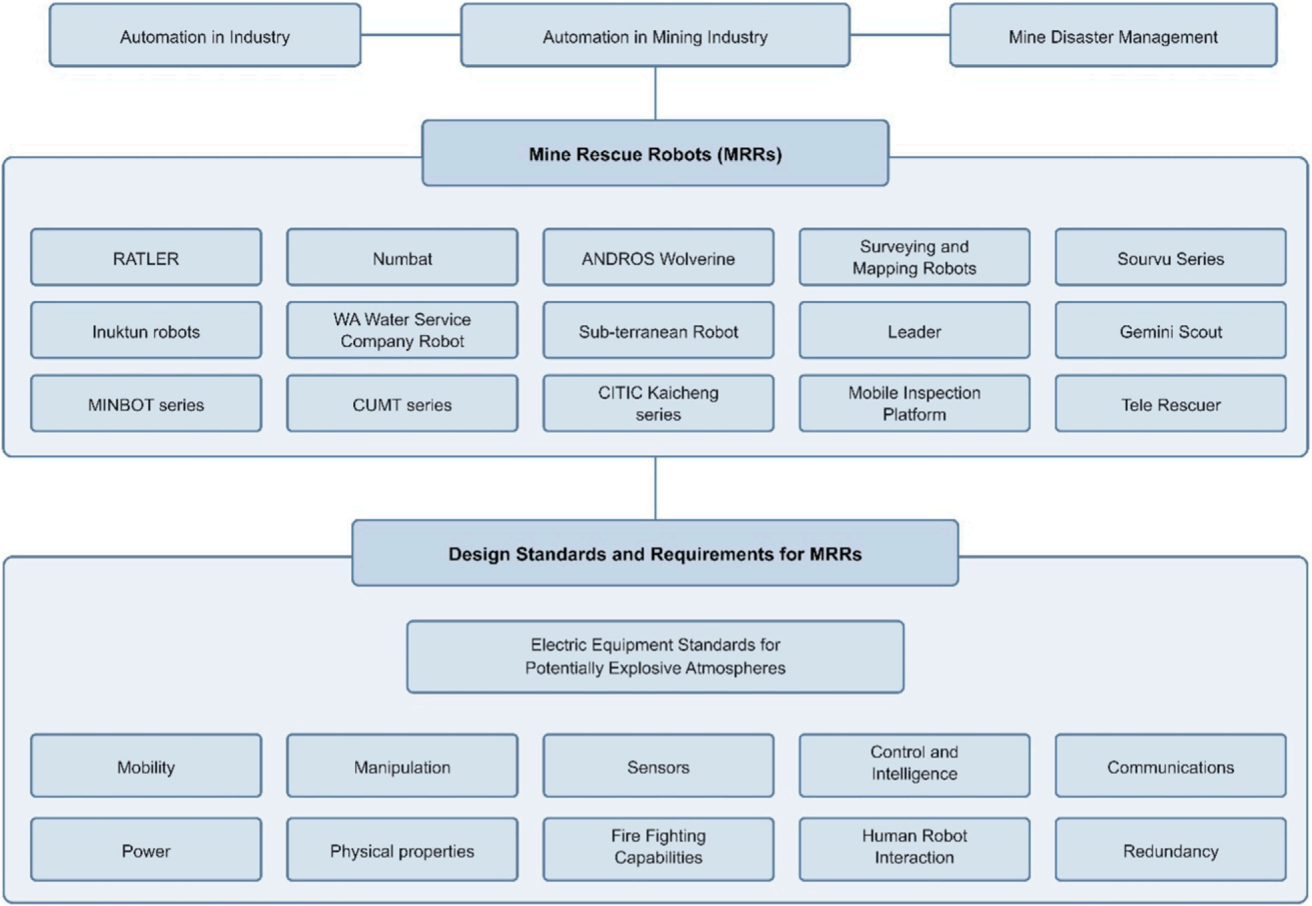
Schematic overview of review structure.

The rest of the article is structured as follows (see also Figure 1): Section 2 discusses the increasing deployment of automation and robotics in the mining industry, alongside the potential of robotic deployments in post-disaster mine search and rescue operations. In Section 3, 15 documented coal-mine search and rescue platforms are presented, while Section 4 reviews the platforms’ design elements. Subsequently, Section 5 discusses the interdependence of design elements, design standards, and trade-offs, as well as emerging technologies and research directions that can advance disaster robotics for mines. Finally, Section 6 discusses the main takeaways from this review and presents the conclusions.

## 2 Automation in mining engineering

Coalitions of mining companies, equipment manufacturers, and software developers have driven automation across the production cycle, motivated by gains in productivity, efficiency, and worker safety. Autonomous systems now operate across all major phases of the mining workflow, including autonomous haulage, load-haul-dump (LHD) machines, longwall shearers and plows, drilling and tunnel-boring systems, dragline swing mechanisms, and tele-remote ship-loading. Combined with the supporting infrastructure of GIS/GPS systems, programmable logic controllers and off-site control rooms have become widespread in the mining industry (Cosbey et al., 2016; Ralston et al., 2014). These deployments relocate operators from active equipment to remote supervision, reducing exposure to underground hazards (Lööw and Nygren, 2019; Lööw, 2020). The strong motivations behind these projects stem from the potential outcomes of delegating parts of the mining process from humans to machines, which can positively impact various economic, productivity, and safety requirements. The results include improved efficiency and accuracy of operations, energy and fuel consumption optimization, reduced maintenance downtime and costs, reduced weather- or human-related interruptions to mining operations, and improved inventory and logistics efficiency through smart monitoring of equipment, extracted material, supplies, and efficient mapping of mines and mine face/geometry progress.

Additionally, automation can drastically improve the health and safety of the mine workers. Although safety statistics have improved in mining, they have not improved as much as in other industries. In 2007, the mining industry had the most work-related health problems in the EU (EUROSTAT Statistical Books, 2010). According to the same study, the number of work-related health problems has increased more in the mining industry than in other sectors. In the US, a series of unexpectedly large-scale mine emergencies in the period of 2006–2010 exposed the mining sector’s neglect of improving regulations, training, and policies regarding safety and health training and emergency preparedness. In the Sago Mine disaster, 12 of 13 trapped miners died after a blast and collapse incident, whereas in the Upper Big Branch Mine disaster, 29 of 31 miners died in an explosion. In both cases, the investigations concluded that substantial safety violations were critical in these incidents (NIOSH, 2023; Davis et al., 2015; MSHA et al., 2006; MSHA et al., 2007; McAteer, 2006).

A smart system typically allows the relocation of mine workers to a safe distance from the operating equipment or even to a remote control room outside of the mine. Automatization of equipment or processes, whether fully- or semi-automated, can minimize the exposure of operators and mine workers to unhealthy and hazardous conditions prevalent in a dynamic mine environment: noise, vibrations, excessive heat, dust, poor illumination, soft tissue damage, roof or ribs collapses, trips and falls, fire and explosions emergencies, caught-in or -between (equipment) hazards, fatigue-related accidents (Cosbey et al., 2016; Occupational Safety and Health Administration, 1998; Sammarco et al., 2018). In addition to automating routine mining operations, smart systems can be employed in emergency situations for search and rescue, evacuation path planning, wearable devices for survival and detection assistance, etc. Despite the progress in autonomous routine operations, search and rescue robots for underground mining emergencies have stalled because of the complexity of the factors involved in mine disaster management and the multitude of failure points.

### 2.1 Disaster management in mine emergencies

Disaster management cycles have varying stages that range from immediate response to long-term planning (Delmerico et al., 2019). Murphy has identified four stages of disaster management: prevention, preparation, response, and recovery. The prevention phase involves ensuring adherence to safety regulations, comprehensive training for mine workers, and regular equipment maintenance. Implementing advanced monitoring technologies and conducting thorough risk assessments also play crucial roles in mitigating potential hazards and ensuring the safety of personnel working in mines. In the preparation phase, plans, procedures, and resources are prepared to respond effectively to potential disasters. When a disaster strikes, the response phase is initiated, involving immediate actions to save lives, provide medical assistance, and protect property. Finally, during the recovery phase, efforts focus on restoring the affected community, rebuilding infrastructure, and addressing long-term impacts, both physical and socio-economic. Together, these phases form a comprehensive approach to managing and mitigating the effects of disasters.

**FIGURE 2:**
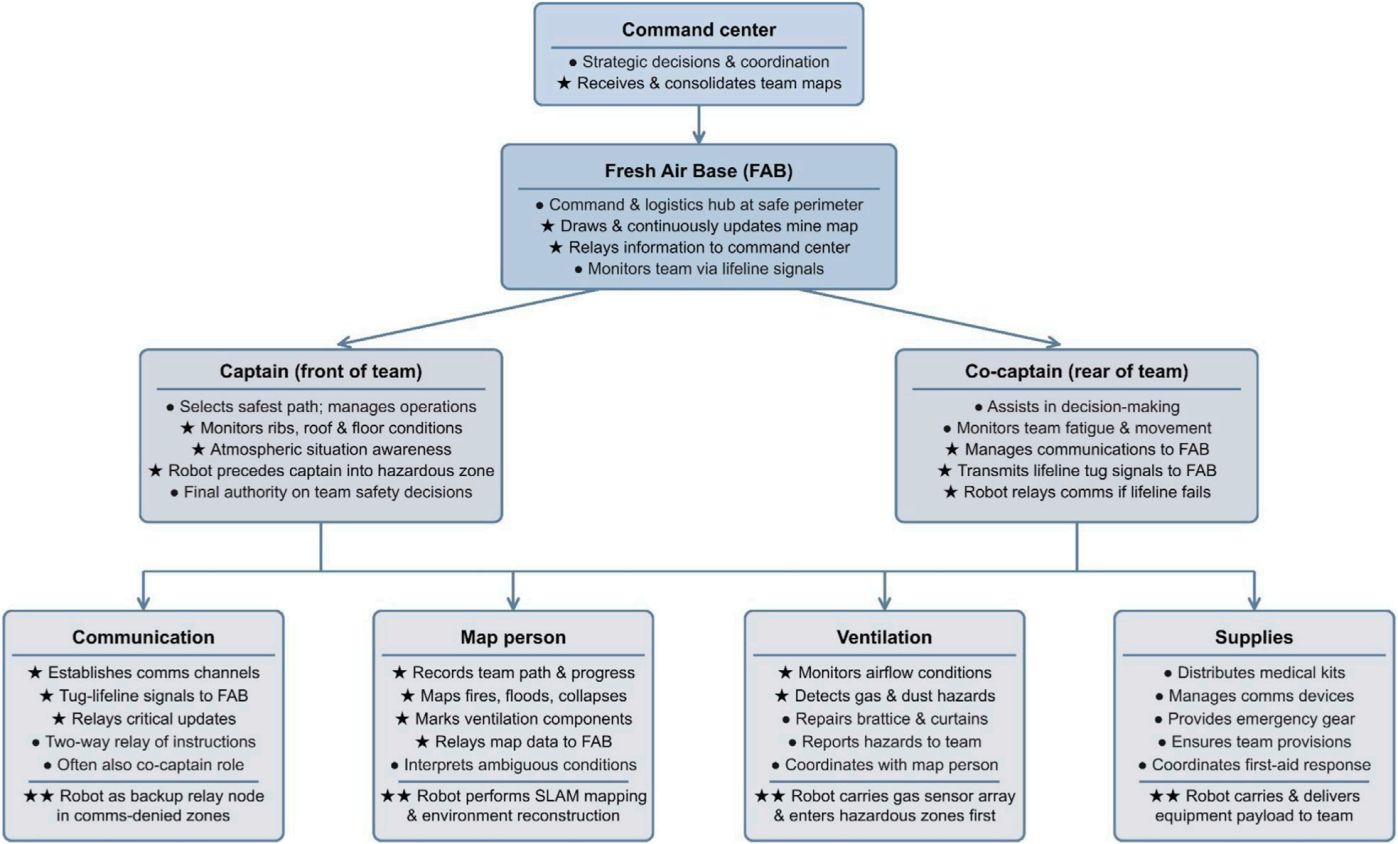
Organizational structure of an underground coal mine rescue team, showing the command hierarchy from the command center through the fresh air base (FAB) to the six operational roles. Responsibilities marked ★ identify tasks for which a CMRR could substitute for or augment human performance; responsibilities marked ● remain inherently human. Regulatory basis: 30 CFR §49.2 (five members and one alternate) and §49.18 (MSHA training requirements).

One of the most demanding tasks in a disaster management cycle is the deployment of a mine rescue team that will undertake a search and rescue operation. Modern mine rescue efforts are highly structured and carried out by proficient teams of trained individuals who collaborate closely. The organizational hierarchy of a mine rescue team, together with the responsibilities of each role and the tasks in which a CMRR can substitute for or augment human performance, is illustrated in Figure 2. Members of underground coal mine rescue teams undergo specific training in accordance with 30 CFR 49.18, which includes advanced mine rescue procedures as prescribed by the Mine Safety and Health Administration (MSHA) Office of Educational Policy and Development (MSHA et al., 2007; MSHA and NMSHA, Advanced Mine Rescue Training (Coal Mines) 2013). According to 30 CFR §49.2, each team shall consist of five members and one alternate, fully qualified, trained, and equipped for emergency mine rescue service.

A typical S&R operation is led by the *captain*, responsible for selecting the safest path and actions based on conditions of ribs, roof, and floor and the data provided by the other team members. The *co-captain* shares leadership responsibilities with the *captain*, assisting in decision making and monitoring the team’s activities. Often, the *co-captain* remains in the rear of the team to detect signs of fatigue or trouble in moving in with the other members. The two captains play a vital role in navigating the mine layout, ensuring communication and safety, and managing the operation effectively. The *communication* person maintains communication channels between the team and the fresh air base (FAB) via battery-powered equipment or, when inoperable, via predefined lifeline tug signals. The *communication* person must ensure the effective two-way relay of critical information, updates, and instructions, facilitating coordination and timely decision making. The *communication* person is often the *co-captain* who remains at the rear of the team. The *map* person records the team’s path and critical mine conditions encountered by the team (e.g., fires, flooding, collapses, ventilation components). These elements are relayed to the FAB, where a second map is drawn and updated. In turn, the FAB personnel relay the information to the command center for a third map. The remaining members carry out *ventilation* (or *brattice*) activities, medical first-aid assistance, and supplies distribution. Ventilation activities involve monitoring and repair of critical ventilation components to ensure a continuous supply of fresh air to the rescue operation area. Effective communication with the rescue team about ventilation status and potential airflow-related hazards is vital for a successful and safe rescue operation. Supplies distribution activities refer to the distribution of vital equipment and supplies to the team, ensuring they have the necessary provisions like medical kits, communication devices, and emergency gear to effectively support the team’s efforts (DoL and MSHA, 2021).

As illustrated in Figure 2, several of these responsibilities present clear opportunities for robotic substitution or augmentation. A CMRR can precede the captain into uncharted sections to collect atmospheric and structural data before human entry, replace manual paper-based mapping with continuous SLAM-generated digital maps, act as a communications relay node when the lifeline signal fails, monitor gas concentrations and airflow continuously without health risk to the ventilation person, and transport equipment payloads to reduce the physical burden during extended missions. By contrast, the command authority of the captain and co-captain, physical repair of ventilation components, and first-aid coordination remain inherently human responsibilities requiring situational judgment that current robotic systems cannot reliably replicate.

Coal mine disasters may involve gas explosions, dust explosions, flooding, fires, and roof falls. Statistically, gas and dust explosions account for more than 60% of coal mine fatalities (Kowalski-Trakofler et al., 2009; Brnich et al., 2010). A quick implementation of rescue operations is vital following an explosion. Even without an explosion, the accident scene is typically a gas-filled, dynamic, and unstructured environment with many obstacles, creating a hazardous situation for rescuers (Li et al., 2020). Underground mines are, in general, poorly illuminated, making it difficult for both humans and robots to navigate and identify hazards or trapped miners. The prevalence of suspended dust and smoke can further affect visibility, while also imposing breathing hazards for humans. Uneven and muddy surfaces or rubble from collapsed tunnels may impede robot and human movement as well. Moreover, toxic and noxious gases can be prevalent in underground coal mines, exposing humans to severe health hazards or even death. The presence of explosive gases imposes limitations on the utility of electronics on robots that must align with strict permission regulations. Special emphasis must be given to the design of the power sources of a rescue robot to ensure an explosion-proof and waterproof robot that can carry out long missions in an underground environment (Khattak et al., 2020). Additionally, the GPS-denied nature of an underground mining environment excludes the utilization of one of the most powerful localization techniques and necessitates the implementation of alternative techniques for efficient self-localization and mapping (Shahmoradi et al., 2020). Establishing reliable communication lines to facilitate uninterrupted data exchange, both for inter-human and human–robot communication, is another significant challenge in deploying rescue robots in a post-disaster environment (Dinelli et al., 2023; Reddy et al., 2015).

It is widely believed that the technological advances of the last decades in robotics, sensors, and software development can be of significant assistance for disaster management. This is evident from the plethora of relevant research efforts and implementations, both in industry and academia, that spans from the literature that focuses on the development of robots that can traverse unstructured or demolished terrains (Whitman et al., 2017), algorithms for localization and mapping in both surface and subterranean environments (Papachristos et al., 2019), algorithms that can detect humans or other salient features, and effective human–machine interfaces for controlling such robots (Semeraro et al., 2022). Robotic technologies, whether remotely operated vehicles, autonomous agents, assistive devices, or novel control interfaces, offer many possibilities for deployment in real-life environments. The complex underground settings present significant challenges for rescue teams as first responders. For rescue operations, field-deployable technologies must be robust, fast, versatile, and easy to use (Delmerico et al., 2019).

## 3 Robots in coal-mine search and rescue operations

In search and rescue operations (S&R ops), the deployed robots must traverse harsh terrain, map the surroundings, locate humans or distinctive features, collect structural and environmental data, and help humans and recover objects, while relaying the collected information to an established control center. Mining-related disaster robots represent a subset of disaster robotics, but their deployment poses unique challenges (Murphy, 2014).

The remainder of the current section covers existing coal mine rescue robots and recently developed robotic platforms capable of exploring underground environments, focusing primarily on unmanned ground vehicles (UGVs), unmanned aerial vehicles (UAVs), and hybrid robots and multi-agent systems. Bipedal/humanoid designs or unmanned surface and underwater vehicles (USVs and UUVs) are excluded because their applicability in mines is limited. These technologies face challenges in navigating the uneven terrain, confined spaces, and complex underground networks encountered in mining environments. Additionally, their specialized functionalities may hinder practicality and versatility when compared to more traditional robotic systems. While technological advancements and niche applications may broaden their potential in the future, their current limitations justify their exclusion from the review.

Table 3 provides a comparative overview of the key operational characteristics of the reviewed CMRRs, including perception sensors, autonomy level, communication channel, explosion-proof and waterproof ratings, and documented search-and-rescue deployment. The table enables a cross-platform comparison of these attributes and serves as a reference point for the design-requirement discussion in Section 5.

**TABLE 3:** Characteristics of mine rescue robots (note: the KQR48 CMRR is not included due to a lack of available information).

CMRR	Country (year)	Perception sensor	Autonomy level	Comms	Explosion-proof	Waterproof	S&R deployed
RATLER (Krotkov et al., 1994; Klarer, 1993; Purvis and Klarer, 1994)	USA (1991–95)	• Gas detection sensors • IR camera • 3 CCD cameras (RATLER II only) • Compass • 3 inclinometers • Gyroscope • Radio frequency transmitter	Teleoperated	Wireless	(unknown)	(unknown)	No
Numbat (Ralston et al., 2001; Hainsworth, 2001; Ralston and Hainsworth, 1998)	Australia (1990–2001)	• Gas detection sensors • 4 video cameras	Teleoperated	Cable (12 km)	Yes	Yes	No
ANDROS Wolverine (Murphy et al., 2009; Li et al., 2020)	United States (2001–2006)	• Gas detection sensors • Surveillance cameras • Night vision cameras	Teleoperated	Cable (1.5 km)	Yes	(unknown)	Yes (7 ops)
Groundhog and Cave Crawler (Zhai et al., 2020; Morris et al., 2006; Baker et al., 2004; Thrun et al., 2004)	United States (c. 2004–2006)	• Gas detection sensors • 2 laser scanners • Night vision camera • Sinkage switches • Gyroscope • Wheel encoders • Tilt sensors	Fully automatic	Wireless	Yes	No	No
Souryu-V (Arai et al., 2008; Masayuki et al., 2004)	Japan (2000–2010)	• Gas detection sensors • Color camera • Infrared camera	Teleoperated	Wireless	(unknown)	Yes	No
Inuktun VGTV (Murphy et al., 2009; Casper et al., 2004)	Canada (2000s)	• Color camera • Color pan-and-tilt camera (with IR light)	Teleoperated	Cable (1.5 km)	No	Yes	Yes (2 ops)
WA Water Service Company Robot (Wang et al., 2018; Reuters, 2010; McDonald, 2023)	W. Australia (c. 2010)	• Gas detection sensors • Vision camera	Teleoperated	Cable (6 km)	Yes	No	Yes (destroyed during mission)
Subterranean Robot (Ray et al., 2009; Maity et al., 2013)	India (early 2010s)	• Sonar • Camera • Compass • Moisture sensor	Teleoperated	Wireless	(unknown)	Yes (UUV up to 10 m)	No
Leader (Wang et al., 2018; RT News, 2016)	Russia (c. 2016)	• Gas detection sensors • Vision cameras	Teleoperated	(unknown)	(unknown)	(unknown)	Yes (no known details)
Gemini-Scout (Reddy et al., 2015; Salton et al., 2017; Callow, 2018)	United States (2008–2016)	• Gas detection sensors • Temperature sensors • Color pan-and-tilt camera (with light) • 2 (additional) color cameras • Thermal imaging camera (IR)	Teleoperated	Wireless and cable	Yes	Yes (up to 45 cm deep water bodies)	No (although NIOSH permissible)
MINBOT-II (Wang et al., 2014)	China (c. 2014)	• Gas detection sensors • Temperature sensor • Humidity sensor • Wind speed • 4 vision cameras	Teleoperated Semi-automatic Fully automatic	Cable (1 km)	Yes	Yes (IP67)	No
CUMT-V (Li et al., 2020; Zhao et al., 2017; Wang et al., 2018)	China (2006–2016)	• CD10 multi-parameter gas sensor • Multi-altitude CH_4_ sensor • 3D LiDAR • RGBD depth camera • IMU • UWB range finder	Teleoperated Fully automatic	Wireless (50 m) and cable (1 km)	Yes	Yes (IP67)	No
Mobile Inspection Platform (Novák et al., 2018; Bołoz and Biały, 2020; Kasprzyczak et al., 2016)	Polland (2008–2016)	• Gas detection sensors • Temperature and humidity sensors • 2 monochrome cameras • RGB camera • Thermal camera (IR)	Teleoperated	Wireless and cable (1 km)	Yes	Yes	No
TeleRescuer (Novák et al., 2018; Cyran et al., 2018; Moczulski et al., 2014)	Poland, Czech Republic, Austria, and Spain (2014–2017)	• Gas detection sensors • Temperature and humidity sensors • Airflow sensors • Rotating 2D LiDAR (effectively 3D) • Pair of stereoscopic cameras • Wide field camera • Rear-view camera • IMU	Teleoperated	Cable (2 km) and wireless (backup system, through deployable nodes)	Yes	Yes	No (although achieved ATEX requirement Group I, Category M1)

### 3.1 RATLER

**FIGURE 3:**
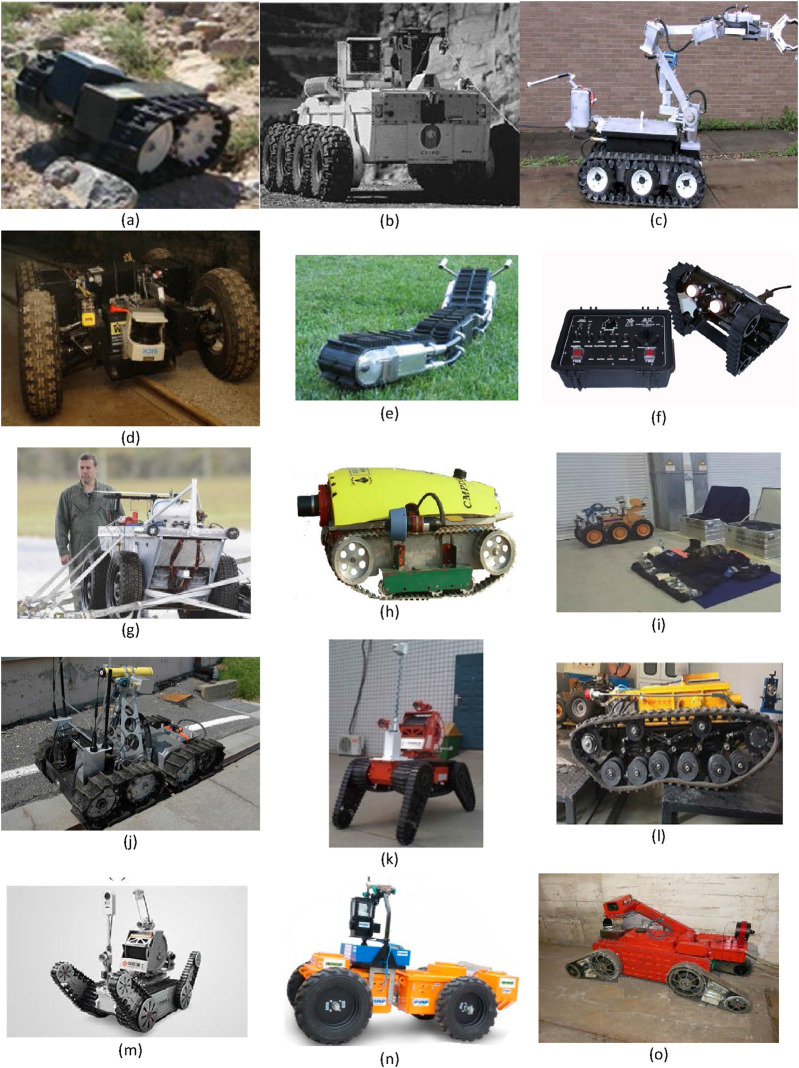
**(a)** RATLER (adapted with permission from Zhai et al., 2020, licensed under CC BY 4.0), **(b)** Numbat (adapted with permission from Zhai et al., 2020, licensed under CC BY 4.0), **(c)** ANDROS Wolverine (V2) (reprinted with permission from Murphy et al., 2009, Copyright © 2009 IEEE), **(d)** Cave Crawler (reprinted with permission from Morris et al., 2006, Copyright © 2006 John Wiley and Sons), **(e)** Souryu-V (adapted with permission from Arai et al., 2008, licensed under CC BY-NC), **(f)** Inuktun VGTV (adapted with permission from Casper et al., 2004, licensed under CC BY 4.0), **(g)** Western Australia Water Service Company Robot (adapted with permission from Wang et al., 2018, licensed under CC BY 4.0), **(h)** Subterranean Robot (reprinted from Ray et al., 2009, with permission of the corresponding author granted via email correspondence), **(i)** Leader (adapted with permission from Wang et al., 2018, licensed under CC BY 4.0), **(j)** Gemini-Scout (reprinted with permission from Reddy et al., 2015, licensed under CC BY-NC-ND), **(k)** MINBOT-II (reprinted with permission from Wang et al., 2014, Copyright © 2014 John Wiley and Sons), **(l)** CUMT-V (B) (adapted with permission from Zhai et al., 2020, licensed under CC BY 4.0), **(m)** KRZ-I (adapted with permission from Zhai et al., 2020, licensed under CC BY 4.0), **(n)** Mobile Inspection Platform (adapted with permission from Bołoz and Biały, 2020, licensed under CC BY 4.0), and **(o)** TeleRescuer (adapted with permission form Novák et al., 2018, licensed under CC BY 4.0).

The Robotic All-Terrain Lunar Exploration Rover (RATLER), the world’s first CMRR, was developed in the United States by a coalition of the Mine Safety and Health Administration (MSHA) and Sandia National Laboratory. The last reported version of RATLER is a dual-body, remotely controlled UGV that is equipped with a radio frequency transmitter, a compass, three inclinometers, a gyroscope, three CCD cameras for stereo mapping, and gas detection sensors (Figure 3a) (Krotkov et al., 1994; Klarer, 1993; Purvis and Klarer, 1994). However, the operation range of RATLER is limited to approximately 76 m. During a field test conducted in December 1998, the RATLER robot failed to meet rescue requirements, particularly regarding maneuverability and remote control distance (Li et al., 2020; Wang et al., 2018; Zhai et al., 2020).

### 3.2 Numbat

The Australian Commonwealth Scientific and Industrial Research Organization (CSIRO) developed the Numbat for coal mine emergencies in the 1990s. It is a high-speed, eight-wheeled UGV with a durable power source that is equipped with four video cameras, gas detection sensors (CH_4_, CO, CO_2_, and H_2_), and sensors to monitor the vehicle, hardware, and power state. Additionally, the platform monitors temperature, atmospheric pressure, and ventilation air speed (Figure 3b). All the information is displayed in the control center on the surface to navigate and inspect a potentially hazardous underground mine. An explosion-proof design keeps Numbat’s components sealed from the potentially explosive atmosphere of a mine within a sealed, nitrogen-pressurized compartment. The robot is controlled remotely with a joystick and a dedicated GUI through fiber-optic cables (Ralston et al., 2001; Hainsworth, 2001; Ralston and Hainsworth, 1998). The platform has been deployed on several occasions in response to underground mine incidents, primarily to provide forward survey capability prior to entry by mine rescue personnel, but it has not been required for personnel rescue (J. Ralston, personal communication, 4 August 2024).

### 3.3 ANDROS Wolverine (V2)

The ANDROS Wolverine Variant-2 (V2), developed by MSHA and Remotec (United States), is a mine-permissible, track-based UGV (crawler) that features surveillance cameras, lighting systems, gas detection sensors, night vision cameras, two-way voice communication systems, and robotic arms (Figure 3c). The V2 robot is explosion-proof and utilizes optical fiber communication to enable communication over distances of up to 1.5 km. However, its heavyweight design and the reliance on wired communications and data transfer (fiber-optic cable) have proved a major disadvantage in the robot’s deployments. MSHA has deployed V2 to seven operations, including two rescue and five mine recovery operations, between 2001 and 2006. Although the robot managed to enter the mine and sufficiently complete the primary mission in four of the seven cases, it had to be manually recovered in two of these successful operations (Murphy et al., 2009; Li et al., 2020).

### 3.4 Surveying and mapping robots

Carnegie Mellon University (United States) developed the Groundhog robot for search missions in underground coal mines. Groundhog is a fully autonomous, wheeled UGV designed for reconstructing 3D maps of abandoned underground mines based on data collected with two laser scanners and a night vision camera. Additionally, the robot is equipped with gas sensors and sinkage switches, a gyroscope, encoders, and tilt sensors. The first successful map of an abandoned mine was acquired from Groundhog in 2003. The robot’s design allows for small-packet, two-way wireless communication with the surface. The successor of Groundhog, Cave Crawler, is equipped with a similar sensor suite and exhibits superior performance, maneuverability, and stability compared to its predecessor (Figure 1d) (Zhai et al., 2020; Morris et al., 2006; Baker et al., 2004; Thrun et al., 2004). However, the design of these robots has focused mostly on the navigation, surveying, and mapping software, and not mine-permissibility issues regarding explosive-proof or waterproof design.

### 3.5 Souryu series

The Souryu I–V robots were developed by the Tokyo Institute of Technology in coalition with the International Rescue System Institute and TOPY Industries, Ltd. (Japan) during the 2000s. The research and experiments on the design of these robots yielded a series of serpentine tracked crawlers that can traverse rubble and detect trapped people in collapsed buildings. Souryu-IV and Souryu-V, the latest and most advanced installments of the series, are equipped with color and infrared cameras and LED lighting and are remotely teleoperated based on the input from the cameras (Figure 3e). Although Souryu-III was equipped with gas sensors, the subsequent models lack gas detection sensors. However, the authors believe that the design of the models allows for mounting additional small sensors. Moreover, the mechanisms are designed to provide water and dust protection. Extensive terrain traversability, locomotion control, and teleoperation tests have shown promising results. However, the authors point out that there is room for improvement regarding stability, the lack of sensory input, and the difficulty of teleoperation of a small-sized robot. These robots have not been reported to participate in active search and rescue operations (Arai et al., 2008; Masayuki et al., 2004).

### 3.6 Inuktun robots

Inuktun Services Ltd. (Canada) has developed a series of pipe inspection and urban search and rescue crawlers. These robots, specialized in accessing human-denied spaces, are tracked UGV robots that are characterized by their small size, with the ability to traverse harsh terrain or hang on pipes. The robots are small-sized, waterproof, tethered, and teleoperated. The Inuktun Micro-Tracks and Inuktun Micro-VGTV (variable geometry tracked vehicle) are equipped with a microphone, a speaker, a motor-driven manual-focus CCD camera, and a camera tilt unit that incorporates halogen lighting (Figure 3f) (Casper et al., 2004). MSHA deployed Inuktun VGTV in Newmont’s Midas Gold Mine (Nevada) rescue in 2007. Although the robot successfully traversed the vertical void and reached the desired site, it was unable to locate the missing miner due to a lack of sufficient light for the cameras. In the same year, a variant of the series named Inuktun Mine Cave Crawler (MCC) was deployed at the Crandall Canyon Mine (Utah). The teleoperated platform was lowered into the mine through a borehole and reached the desired floor after a few attempts. The main challenge was to keep the camera lenses clean during the descent. MCC explored a small part of the collapsed mine, showing the size of the collapse. It was decided to abort and retrieve the robot, but the robot was lost due to falling debris (Murphy et al., 2009; Murphy, 2010).

### 3.7 Western Australia Water Service Company robot

The Western Australia Water Service Company provided a robot to a rescue mission deployed at the Pike River coal mine (Greymouth, New Zealand) after an explosion trapped 29 miners in November 2010. Although this robot was designed for pipeline operations, it was retrofitted and deployed as a CMRR. This robot was a wheeled UGV equipped with gas detection sensors, a camera, and lights, while a 6-km-long fiber-optic cable allowed for data transmission and teleoperation (Figure 3g). Two more robots (bomb disposal robots) were deployed in the same mine rescue mission before the Australian robot. The first robot was a robot provided by the New Zealand Defense Force, which short-circuited before traveling more than 1 km, while the second robot also stopped operating long after its deployment. All the robots were destroyed in the second explosion before locating any of the trapped miners (Wang et al., 2018; Reuters, 2010; McDonald, 2023).

### 3.8 Subterranean Robot

The Subterranean Robot (SR) is an amphibious robot developed by the Central Mechanical Engineering Research Institute (India) in the early 2010s. The SR can move and collect data on both the ground and underwater. This waterproof hybrid robot combines the abilities of a track-based UGV (also convertible to wheel-based) and a UUV operational up to 10 m and able to resurface automatically. The SR, which is intended for locating trapped miners, is equipped with sonar, camera, compass, and moisture measurement sensors (Figure 3h). The robot is operated remotely (without tether) and transmits sensor data through dual acoustic and radio frequency communications. It can operate mostly autonomously with sporadic steering corrections from the remote operator, who can control the robot and monitor the sensor outputs through a custom GUI. Field tests in an underground coal mine have shown promising results but also path deviations due to sensor data shortage and robot-floor slippage (Ray et al., 2009; Maity et al., 2013). The robot has not been reported to have been deployed operationally.

### 3.9 Leader

A CMRR developed by the Russian Special Forces has been reported to actively participate in a search and rescue operation after three gas explosions occurred in a Theo Kuta Mining Company coal mine near the city of Vorkuta, Komi Republic, Russia, in 2016. The blasts killed 36 miners, including the first-response, five-member rescue team. The Leader robot is a remotely controlled, eight-wheeled UGV equipped with cameras and gas sensors (further details are unknown) (Figure 3i). The principal (known) capability of the robot was to inspect the mine and assess the viability of sending a second rescue team into the collapsed mine (Wang et al., 2018; RT News, 2016).

### 3.10 Gemini-Scout

Sandia National Laboratory and the National Institute for Occupational Safety and Health (NIOSH, United States) collaborated for the development of the Gemini-Scout in 2008. It is equipped to handle any number of obstacles, including rubble piles and flooded rooms, to help rescuers reach trapped miners safely and efficiently. Gemini-Scout is a remotely controlled, rubber-tracked dual-body UGV specifically designed for coal mines. It is equipped with gas and temperature sensors, a color pan-and-tilt camera with light, two additional color cameras, and a thermal camera (infrared) collecting data that are transmitted wirelessly to the operators. Its design allows for great mobility in rough terrain, while it is explosion-proof, partially waterproof (up to 450 mm), and permissible for coal mines (Figure 3j). However, the robot has never been operationally deployed in a rescue (Green, 2013; Reddy et al., 2015; Salton et al., 2017; Callow, 2018; Deuel et al., 2018).

### 3.11 MINBOT series

The Harbin Institute of Technology (China) developed the MINBOT-I and MINBOT-II robots, with the latter being an improved version of the former. The MINBOT-II robot is a modular, track-based UGV that can be (manually) configured with additional swing arms for improved mobility and obstacle surmounting. The robot is equipped with gas and environmental sensors for CH_4_, CO, O_2_, temperature, humidity, and wind speed, as well as four vision cameras, two-way audio, and infrared lights. The data are transmitted through an optical fiber cable with a maximum length of 1 km (Wang et al., 2014). Emphasis has been given to harsh terrain mobility and obstacle-tackling design, as well as to waterproof, explosion-proof, and intrinsically safe design. MINBOT-II can be deployed through telecontrol, fully automatic, or semi-automatic modes (Figure 3k). Wang et al. (2014) present their thorough investigation of the design and experimental testing of the track system, the suspension, the communications, the power supply, and the mobility and path planning modules. This robot has not been reported to participate actively in any search and rescue operations.

### 3.12 CUMT series

A series of CMRRs has been developed by several Chinese universities and research units. China University of Mining and Technology has been developing the CUMT series robots since 2004. The series started with the CUMT-I in 2006 (the first CMRR of China), with the triplet of CUMT-V (A-C) as the latest (fifth) installment developed in 2019 [32]. CUMT-I is a remotely controlled, track-based UGV that can transmit video, detect gases, and move small obstacles, but it is not explosion proof and does not perform well in terms of mobility and perception. As a result, it has never been operationally deployed because it does not fulfill rescue requirements (Wang et al., 2018; Li et al., 2022).

The technology and design aspects of explosion-proof, walking, communication, sensing, localization, navigation, and motion control techniques have been gradually improved with each new product of the series. CUMT-V (C) is equipped with 3D LiDAR, an RGBD depth camera, an inertial measurement unit (IMU), and an ultra-wideband (UWB) range finder used for navigation and mapping (Figure 3l). Additionally, the robot carries “one intrinsically safe audio, one intrinsically safe microphone, and one intrinsically safe CD10 multi-parameter (10 parameters) gas sensor,” and a movable CH_4_ sensor for multi-altitude gas concentration detection (Li et al., 2022). The robot can be deployed in teleoperation (primarily for detection, rescue, and mapping missions) or fully autonomous (primarily for inspection tasks) modes. A combination of three robots equipped with Wi-Fi and fiber-optic cable (1 km long) communications technology is used with the aim of a minimum of 3 km of reliable communications. Moreover, CUMT-V C carries a small amount of fire extinguishing material for active firefighting. A custom GUI allows a user to remotely control all three robots simultaneously and monitor the sensor feedback in real time (Li et al., 2020).

The development of the CUMT robot series has focused on mine-permissible materials and designs, and the CUMT-V, B and C robots have acquired the Mining Products Safety Approval and Certification (MA Certification). Although the robots have not participated in any mine rescue operations, a series of physical tests have proved that the CUMT-V (B-C) robots can handle practical applications of coal mine rescue and firefighting (Li et al., 2020; Wang et al., 2018). These robots were later renamed as mine search-and-rescue robots (MSRBOTs) (Zhao et al., 2017).

### 3.13 CITIC Kaicheng series

The Chinese Tangshan Kaicheng Electric Company has developed a series of MRRs (see Table 2 of Wang et al., 2018) with the KQR48 robot succeeding in obtaining the Mining Products Safety Approval and Certification (MA Certification), which permits the robot to enter a mine. Although details of the robot’s design are not published, it is known that the robot has not yet been deployed operationally (Wang et al., 2018).

KRZ-I is another of the company’s safety- and explosion-proof certified robots for mine search and rescue. It is equipped with a camera assisted by infrared lights and gas sensors and can carry rescue tools and supplies (Figure 3m). Integrated posture and obstacle avoidance intelligence enable the robot to operate without compromising its safety (Zhai et al., 2020). It is not known whether the robot has been deployed operationally.

### 3.14 Mobile Inspection Platform

A consortium of the Institute of Innovative Techniques EMAG and the Industrial Institute of Automation and Measurements PIAP (Poland) developed the Mobile Inspection Platform (MIP) in 2016. The MIP robot was designed to fulfill the requirements of three European Commission directives regarding machinery in explosion- and fire-hazardous environments: Directive 94/9/EC (ATEX) (EUR-Lex, 2014), Machinery Directive 2006/42/EC (MD), and Directive 2004/108/EC (EMC). The MPI is a four-wheeled, dual-body UGV equipped with gas detection sensors for CO_2_, CH_4_, O_2_, and CO, temperature and humidity sensors, and four cameras and lighting, including two monochrome cameras, an RGB camera, and a thermal camera (Figure 3n). Emphasis has been given to the intrinsically safe design of the MPI that includes a pressurized enclosure for housing the RGB and IR cameras with automatic power cutoff in the case of a pressure drop, intrinsically safe monochrome cameras and lights, explosion-proof motors, flameproof enclosures for housing the motor drives and controllers, electrically conductive tires and wheel rims, etc. The platform supports two-way data and transmits commands through Wi-Fi when on the surface and through an (1 km long) optical fiber cable when in an underground environment (Novák et al., 2018; Bołoz and Biały, 2020; Kasprzyczak et al., 2016). A major disadvantage of the platform is its size and weight. Although extensive functional and traction tests have been conducted in training excavations of the Central Mines Rescue Station (Bytom, Poland), the CMRR has not been reported to participate in an actual rescue mission.

### 3.15 TeleRescuer

An international consortium between the Silesian University of Technology (Poland), the Vysoka Skola Banska-Technical University of Ostrava (Czech Republic), the Universidad Carlos III de Madrid (Spain), COPEX (Poland), SIMmersion GmbH (Austria), and Skytech Research (Poland) during 2014–2017 developed the TeleRescuer robot. The CMRR is built in accordance with Directive 94/9/EC (ATEX) (EUR-Lex, 2014), which regulates the manufacturing and operation of equipment in explosive-hazardous atmospheres in the European Union, and the IEC 60079 Series Explosive Atmosphere Standards. The TeleRescuer is a teleoperated UGV with four flipper-tracked arms for motion. The robot is equipped with a 2D LiDAR sensor mounted on a rotating axis that supports 3D mapping. The TeleRescuer has an extendable and tiltable arm that carries a pair of stereoscopic cameras, a wide-field camera, a rear-view camera, and a thermal camera, and sensors that measure various gas concentrations, airflow, relative humidity and temperature, an inertial measurement unit (IMU), and LED lights (Figure 3o). The design of this CMRR integrates flameproof enclosures and enclosures with methane detectors for automatic power cutoff to ensure mechanical and electrical protection of its systems. The sensor data and the teleoperator commands are transmitted through an optical fiber cable. Although lighter than other CMRRs, the robot’s size is not ideal, and its operation range is limited to 2 km (length of the fiber optical cable). The TeleRescuer has been extensively tested in both simulated and real conditions, as well as in a training coal mine, Queen Luiza (Zabrze, Poland), but has not been deployed operationally (Novák et al., 2018; Cyran et al., 2018; Moczulski et al., 2014; Kot et al., 2017).

## 4 Design standards and requirements for CMRRs

Specialized standards that regulate the design, testing, and deployment of robotic platforms in mining environments have not yet been established (to the best of the authors’ knowledge). However, a series of international and local standards have evolved regarding the use of electrical equipment in potentially explosive atmospheres. As discussed above, these standards have been incorporated into the design of various CMRRs to ensure appropriate and safe selection and assembly of different hardware components.

A complete absence of standardization that would define computational and functional performance requirements is evident. This could be attributed to the relatively young age of the field of robotics, especially in the mining sector, and therefore the lack of cumulative experience in deploying robotics in the mining sector. As a result, researchers and manufacturers in the field of applied robotics must exercise their individual judgment to define the appropriate computational and functional capabilities of robotic platforms. A consensus regulating the design, testing, and deployment of robotic platforms in mining environments could guide the manufacturers and operators in developing reliable and robust rescue and disaster robotics.

### 4.1 Electric equipment standards

The design and utilization of machinery and electronics in mines is regulated by various regulatory acts and standards to ensure robust and safe deployment. Emphasis is given to the potential for explosions caused by malpractice and malfunction of machinery and electronics used in mines because of the prevalence of potentially explosive atmospheres. Although different countries have established their individual regulations and standards, these standards usually align with the IEC 60079 Series Explosive Atmosphere Standards (International Electrotechnical Commission) (Novák et al., 2018; IEC, 2017). Supplementary Appendix Table A1 in Appendix A lists the IEC standards series parts and the topics they cover. The standards also define hazardous area classifications, equipment and protective system type selection, general safety technical requirements, basic safety requirements for equipment in explosive environments, design and structure of protective systems, and inspection and assessment procedures. Specifically, the IEC 60079 standards outline comprehensive guidelines related to equipment and components designed for use in explosive atmospheres. They cover various protection types, including flameproof enclosure, pressurized enclosures, power filling, liquid immersion, intrinsically safe systems, and equipment design, selection, installation, maintenance, and repair for different hazardous environments, gas, and dust atmospheres. Additionally, they include specifications for gas detectors, electrostatic hazards, and cap lights for mining, ensuring the safety and compliance of equipment used in potentially explosive settings.

In the European Union, the European Commission Directive 2014/34/EU, also known as ATEX, is the primary legislation for this purpose. ATEX addresses various critical aspects, including the obligations of manufacturers and operators, essential health and safety requirements, conformity assessment procedures, and the control of products in potentially explosive environments. These standards cover mechanical strength, electromagnetic and ultrasonic energy emissions, thermal endurance, and the safe incorporation of cells and batteries in gas-explosive atmospheres (EUR-Lex, 2014). In North America, the potentially dangerous areas are classified with the Hazardous Locations (abbr. HazLoc) classification. The associated HazLoc product standard defines the requirements for equipment used in these potentially hazardous areas. In China, the Guo Biao (GB) system uses national standards issued by the Standardization Administration of China (SAC), with GB3836 standards regulating applications in explosive atmospheres. These standards are identical to the IEC 60079 standards (IEC, 2017). The Russian Federation and neighboring countries use the Technical Regulations CU TR012/2011 (EAC certification for the Eurasian Union, 2011). In Brazil, INMETRO Ordinance No. 83 (published on 03 April 2006) regulates the design, acquisition, construction, assembly, and conditioning of electrical installations and equipment for potentially explosive atmospheres involving flammable gases and vapors. Finally, Australia uses the NSW Coal Mine Health and Safety Regulation.

### 4.2 Common design elements for CMRRs

The physical, functional, and computational capabilities of the existing CMRRs or robotic systems able to navigate in harsh, subterranean environments vary greatly based on the objectives and the judgment of the researchers/manufacturers. Moreover, the objectives and performance metrics of the capabilities of disaster robots are affected by the constant advancement of hardware and software solutions. At the same time, considering the limited experience of deploying disaster robots in mine or other emergency situations, the study of disaster robotics deployments in different environments and scenarios will push the frontier on the capabilities and the minimum requirements for CMRRs.

The examination of recent CMRR deployments, as documented in international literature, outlines shared objectives and requirements. For example, Murphy et al. (Murphy et al., 2009) define (and rank by importance) the requirements for CMRRs classified into eight principal areas of robotic features. Two additional areas are deemed important to be added by the authors based on the trends exhibited in more recent literature.

The ten design elements common to CMRRs, namely, mobility, manipulation, sensors, control and intelligence, communications, power, physical properties and permissibility, firefighting capability, human–robot interaction, and redundancy, do not exist in isolation. As illustrated in Figure 3, they form an interconnected network of mutual trade-offs and regulatory constraints in which choices made in one element propagate through several others. A more capable sensor suite demands greater computational resources and increased power, which in turn affects the platform’s physical envelope and endurance; a higher level of autonomy drives bandwidth requirements that are directly bounded by intrinsic safety certification limits; and the permissibility requirements imposed by underground coal mine regulations act as non-negotiable overrides on the physical design, communications hardware, power sources, and sensor enclosures alike. These interdependencies mean that CMRR design cannot be treated as a sequential checklist of independent specifications; rather, it is a simultaneous co-optimization problem in which advancing one element frequently comes at a cost to another. The ten elements are discussed individually in the sub-subsections below, while their mutual trade-offs and the deeper taxonomy of design-requirement categories that structure them are analyzed in Section 5.1.

#### 4.2.1 Mobility

A disaster robot needs adaptable locomotion mechanisms that allow for terrain versatility. Confined spaces, uneven or inclined surfaces, prevalence of water, mud, rubble, etc. are common conditions that a CMRR will encounter in a post-disaster mine. Reliable mobility design will enable robots to navigate complex subterranean environments, surmount obstacles, and swiftly maneuver through confined spaces, thus ensuring effective exploration, survivor detection, and response capabilities within the challenging conditions of underground mines. According to Murphy et al. (2009), minimum mobility requirements include the ability to “move forward, backward, turn left/right in all environmental conditions,” while the ability to “transition between vertical to horizontal mobility” is also a high-value capability.

The use of tracked or wheel-based locomotion is the primary decision when designing a disaster robot. A muddy terrain dispersed with rubble necessitates the utilization of wheeled robots (e.g., Numbat, Leader, MPI, and TeleRescuer), track-based locomotion (e.g., Souryu-V and MINBOT-II), or wheel-tracked locomotion (e.g., RATLER, ANDROS V2, Gemini-Scout, and CUMT-V), which allow for heavy robots to effectively move over highly uneven terrain. In the first case, designs with more than four wheels are favorable, like Numbat (eight wheels) and Leader (six wheels). Alternative solutions that enhance traction, stability, obstacle crossing, slope climbing, stair climbing, and trench-crossing capabilities include all-wheel drive (AWD), multi-body chassis connected with flexible joints (RATLER, Souryu-V, Gemini-Scout, and MPI), and swing arms (MINBOT-II and TeleRescuer). The design and testing of joints and swing arms require extensive simulations and testing to ensure selection of proper materials and dimensions (Wang et al., 2014).

Mobility performance is tested with respect to various parameters. For example, Dinelli et al. (2023) conducted comprehensive motion behavior testing of five different walking mechanisms for the CUMT-V robot to optimize basic performance parameters such as maximum walking distance, speed and climbing angle, maximum obstacle height, trench width, and continuous step capability. Furthermore, as IEC (2017) emphasized for the design of the MINBOT series, the mechanical structure and the suspension parts of the mobility system must be designed considering the debris and gravel present in a rough post-disaster, off-road environment, and the excessive vibrations to which the robot will be exposed.

Additional mobility aspects include tethers, vertical mobility, and speed. Tethers add significant complexity to a platform (e.g., unwinding/recoil system) and hinder maneuverability through motion restriction and added overload. As an example, the deployment of CUMT-V needs three robots due to the overload of optical fiber cable that exceeds 1 km in length (Li et al., 2020). Vertical mobility would allow a CMRR to enter or navigate around a mine through shafts (Murphy et al., 2009), either by clinging to existing pipelines or by deploying their own descending/ascending cable equipment. Finally, although a disaster robot is not expected to operate at high speeds, it is necessary to be able to achieve reasonable speeds.

#### 4.2.2 Manipulation

Manipulation entails the incorporation of dexterous robotic arms and grippers capable of interacting with objects, tools, doors, or cables. This capability enables the robot to perform intricate tasks such as debris removal, equipment manipulation, opening doors, turning valves, and potentially life-saving actions like providing medical aid or assisting trapped individuals, thereby enhancing the robot’s efficacy in conducting successful search and rescue missions in a post-disaster environment. Although robotic arms have been effectively incorporated in industries like manufacturing, military armament, healthcare, etc., they usually have one end fixed and operate within an unobstructed working space. In recent years, technological advances have enabled collaborative robots, also called cobots, which can work collaboratively with humans in the same working space. Impressive research has been done with anthropoids that can handle a variety of tasks with articulated arms. A notable example is the DARPA Robotics Challenge with the objective of motivating and enhancing research on socially assistive robotics (SAR) that can rescue and aid workers in disaster response operations. These robots were principally bipedal and dual-armed anthropoids (Pratt and Manzo, 2013). The competition track required robots to complete a series of tasks in a human-engineered environment that required efficient and timely handling of doors, valves, tools, and even vehicles. Of 22 robots from five different countries, the winner of the challenge was team KAIST, which designed the RC-HUBO humanoid. RC-HUBO can transition from bipedal walking to wheeled rolling when kneeling (Jung et al., 2018). Even though Murphy et al. (2009) believed that a robotic arm on a CMRR need not “… be capable of pulling or pushing objects, such as removing rubble,” this capability could be critical in S&R operations. ANDROS Wolverine V2 is a CMRR equipped with a robotic arm and gripper, and as reported, “the robot was able to move some debris with its effector” when it was deployed at the Brown Forks mine in August 2004. However, no further information is available for the arm’s performance. TeleRescuer also has an extendable arm, but it is only for assisting sensors to collect data at higher points.

#### 4.2.3 Sensors

Robots that operate in complex environments require many sensors so that the system can effectively sense the surrounding environment. A diverse and well-selected sensor suite plays the most crucial role in automating robots in terms of navigation, exploration and mapping, manipulation of objects, decision making, and self-health monitoring. In general, sensors can be divided into five categories based on the objective of the captured data:

- Mapping sensors (primary): Sensors, usually exteroceptive, that allow a robot to sense the surrounding environment. Commonly, the data from these sensors are fused and processed to create maps and visualizations of the robot’s environment. These maps are the cornerstone of a successful mission for fully- and semi-autonomous robots. Alternatively, remotely controlled robots must relay the raw data to the operator. Typical sensors in this category are a) time-of-flight laser, infrared, and acoustic rangefinders that can be 1D, 2D, or 3D. The most powerful and, hence, popular are the 2D and 3D LiDAR scanners, and b) cameras, including infrared, RGB (i.e., vision), RGBD, night vision, thermal imaging, and monochrome. These sensors produce the most accurate and information-rich data but also exhibit high power consumption and require high computational processing power when used in fully- and semi-autonomous robotics. These sensors provide the principal input of the simultaneous localization and mapping (SLAM) algorithms. Additionally, these sensors have the most potential in detecting and locating humans or landmarks and features of importance.
- Pose sensors (secondary mapping): Sensors, usually interoceptive, that provide information about the pose and incremental movement of the robot. Typical such sensors are inclinometers, inertial measurement units (IMUs), gyroscopes and accelerometers, tilt sensors, GPS units, and wheel encoders. These sensors yield higher frequency and consume less power, although they are more error-prone and are not as information-rich. Their data can be combined in various odometry algorithms that can provide localization of the robot. However, the data are more often integrated with data from the first category of sensors to enhance the accuracy of maps. For example, they can help to close the iterative closest point (ICP) loops, which is a crucial step of the SLAM algorithms.
- Environmental and chemical sensors: Sensors that measure temperature, humidity, water-floating sensors, airflow, wind speed, and gas detection. These relatively simple and low-power-consuming sensors capture information about the environmental conditions of the robot’s surroundings, which, if combined and geolocated with the 2D or 3D maps of the sensors described above, allow for assessing the conditions of the environment. Naturally, this information is invaluable, and hence highly sought after, in emergency situations as it can assess the safety and feasibility of dispatching human rescuers and medics. Gas detection sensors that measure concentrations of CO, CO_2_, CH_4_, O_2_, fire fumes, and smoke are of the utmost importance for mine rescue robotics. Additionally, the sensor data can be directly used in decision making and for switching between robot modes and functions. As an example, water-floating sensors at the bed of a robot can warn a robot about imminent bodies of water and prevent it from submersion into water.
- Machine health sensors: Monitoring the health and functionality of a robotic platform, especially in cases of long missions, is an aspect that developers must consider. Monitoring information such as battery performance, power level, short circuits or sensors, sensor health, and tire and wheel status allows for a robotic platform or a remote operator to assess the robot’s ability to execute the mission at hand, assess the validity and accuracy of the collected data, and identify necessary actions for preventing failure and loss of the platform.
- Communication equipment and sensors: See Section 4.2.5.

All the reviewed MRRs are equipped with monochrome or RGB vision cameras as a primary sensing tool, and thermal, night vision, and depth cameras are often combined with vision cameras. The LiDAR scanners are the second most used primary sensing tools, especially in platforms such as Groundhog, Cave Crawler, ATRV CUMT-V, MPI, and TeleRescuer, which emphasize autonomous mapping. Interoceptive pose sensors are mainly used in combination with LiDAR. Gas detection sensors are indispensable and are present in every MRR reviewed, with temperature and humidity sensors being the second most used environmental/chemical sensor. Audio communication is a common feature in the MRRs implemented with two-way audio or radiofrequency equipment. Notably, the CUMT-V is equipped with intrinsically safe two-way audio systems, while TeleRescuer is the only robot capable of dropping communication nodes that create an *ad hoc* wireless network. Finally, the European MRRs, MPI and TeleRescuer, are the only robots that integrate machine health monitoring sensors because their development has focused significantly on fulfilling the EU electric equipment standards for potential explosive environments.

#### 4.2.4 Control and intelligence

Control of a platform is equivalent to the robot’s software ability and overall intelligence (Murphy et al., 2009). This aspect is closely related to the level of automation. Control requirements include the integration of algorithms, sensory feedback loops, and user interfaces that enable precise manipulation of a teleoperated robot or enable information-rich monitoring of a fully autonomous robot. By developing intuitive and human-centered control systems that accommodate teleoperation, autonomous navigation, and adaptive behaviors, designers empower operators to guide and monitor the robot effectively through complex underground terrains, avoid obstacles, and make informed decisions. Holistic consideration of control mechanisms, human-centered data visualization, and graphical user interfaces ensures the robot’s ability to execute tasks with accuracy and fulfill its mission objectives, thereby enhancing its role as a versatile and reliable tool for successful search and rescue operations in the challenging and dynamic mining environment.

Most of the reviewed MRRs fall into the class of teleoperated robots. These robots are controlled by joysticks and the aid of simple GUIs that visualize the video camera feedback. Groundhog and Cave Crawler are designed with fully autonomous navigation and mapping capabilities. However, it should be noted that these robots are predominantly exploration robots. Of the remaining MRRs, MINBOT-II and CUMT-V are the only platforms capable of fully autonomous operations that incorporate mapping, navigation, and mission-specific decision making. However, both platforms retain the teleoperation functionality that is still a necessary requirement for robots deployed in complex environments. Murphy (Murphy et al., 2009) stated two reasons that require the presence of a human in the loop: “first, a human is needed to interpret imagery and redirect the search; the inability to predict the outcome of a disaster means that it is unlikely to automate any of the visual sensor interpretation tasks. Second, fully autonomous tasking of a robot, that is, where the robot is sent out and then returns with a report, means that if the robot fails, the information collected to that point would be lost.” Although technological advancements during the last decade have made significant leaps in robotics hardware and software, the former reason remains undoubtedly valid, whereas the second reason is partially resolved but still needs careful consideration from the designers. Given the high likelihood of failure, a rescue robot must continuously provide the responders with sensor data. Thus, teleoperation is a critical and necessary requirement. Semi-autonomy can provide a means of combining the benefits of teleoperation and full autonomy for navigation and image interpretation.

#### 4.2.5 Communications

Communication requirements are a challenge for mines and MRRs, and especially for post-disaster environments. There is limited communication in working mines, even in routine operations. The mine may have a few strategically placed wired telephones, but in general, there is no wireless or physical communication infrastructure. It is apparent that in emergency situations, reliable communication is of the essence for robotic rescue platforms. For example, two-way audio is a common functionality for most mine rescue robots, as evidenced in the early 2000s review of Murphy et al. (2009).

A robotic platform cannot rely on the presence or accessibility of such communication infrastructures. Therefore, the design of an MRR should consider solutions by integrating multiple communication systems, such as wireless or high-bandwidth channels, antennas or repeaters, cables, and optic fibers. These systems must facilitate seamless connectivity between the robot, human operators, rescue personnel, and the above-ground command center. The communications system must enable real-time exchange of critical information, remote control, and coordination. Effective communication ensures timely updates on mission progress, allows for remote operation and intervention when necessary, and enhances overall situational awareness, enabling the robot to play a pivotal role in successful search and rescue missions. Regardless of the communication mechanism, the network must have sufficient bandwidth to support concurrent information and video transfer.

In general, communication systems for two-way data transfer can be divided into two main categories, each with distinct advantages and disadvantages: wireless and tethered. Some developers integrate hardware and software systems that allow a robot to drop communication nodes that build *ad hoc* wireless networks in cases where no communication channels are available in a post-disaster environment. Tethered communications are more reliable and can support faster rates of data transfer, but tethers are susceptible to tangling or breakage and limit a robot’s operational range. Wireless communications negate the need for cumbersome and heavy cables, but the expected long transit distances and the need for line-of-sight suggest that the deployment of nodes, antennas, or repeaters will be needed to support a wireless network (Murphy et al., 2009; Li et al., 2022). Both types of communications are popular, as seen by the reviewed MRRs. The platforms CUMT-V, MPI, and TeleRescuer are the only ones that are equipped with both capabilities, while CUMT-V is the only robot that combines wireless and cable communication in a non-mutually exclusive manner.

#### 4.2.6 Power

Efficient energy sources, batteries, and power management systems are a backbone feature for any robot. Power supply and management in MRRs, which need onboard power sources that can sustain long-duration operations, are critical aspects of their design and operation. Reliable power provisioning enhances the robot’s ability to support critical functions such as mobility, communication, sensing, and manipulation, ultimately contributing to the success and effectiveness of rescue operations by extending mission duration and minimizing downtime. Typically, robotic platforms are equipped with onboard batteries that supply the energy for operating the electrical components, such as sensors, microcontrollers (computational capabilities), lights, motors, and actuators. The robot’s operational time, therefore, relies heavily on battery capacity. The battery selection is not a trivial matter because it requires the consideration of multiple factors such as electrical components requirements and energy consumption rates, computational power requirements, and mechanical and mobility requirements. The selection and design of a suitable battery-based power system for an MRR must consider the voltage level, capacity, discharge rate, activation time, charging time, lifespan, and cost efficiency (Dinelli et al., 2023). Additionally, a mining environment introduces another crucial parameter that limits the energy source options: energy sources must align with the strict permissibility regulations and standards. As discussed in the previous section, detailed regulations must be considered for permitting batteries to operate in potentially explosive or flammable atmospheres. Therefore, selecting the optimum batteries that will perform safely in underground rescue applications remains an ongoing challenge. By optimizing power consumption, selecting suitable energy storage solutions, and implementing intelligent charging mechanisms, designers ensure the robot’s sustained operation throughout extended missions.

#### 4.2.7 Physical properties, intrinsic safety, waterproof and explosion-proof designs

The physical properties of a disaster robot must optimize functionality and durability, especially considering the harsh and dynamic environments in which these robots must operate. The design must balance cost versus functionality and transportability and meet the operational expectations (Murphy et al., 2009). The selection and configuration of materials, size, weight distribution, and structural integrity must ensure optimal performance and safety within a harsh underground mine environment. An extensive consideration of physical properties directly influences the robot’s maneuverability, stability, and self-balancing in rugged environments, durability against corrosion, minor rockfalls, moisture and water, and rendering explosion-proof characteristics. By combining intrinsic safety measures (prevention) with waterproof and explosion-proof designs (segregation and containment), search and rescue robots can effectively operate in harsh underground mine environments, while minimizing the risk of causing fires and explosions. However, a holistically optimum design exhibits various complexities because solutions for optimizing one design aspect may conflict and hinder optimization of another design element.

Commonly investigated design aspects include (but are not limited to):

1. Size and weight: The size and weight of an MRR affect maneuverability in constrained spaces, navigation through tight passages, and motor power consumption and transportability of the platform. Increased size and weight can be a disadvantage and hinder the MRR’s functionality or even ability to access a mine (Novák et al., 2018). A significant portion of a robot’s size and weight is attributed to its chassis and mobility system and consequently to the mobility requirements for the operating environment. Harsh environments, like a post-disaster mine site, are characterized by uneven terrain and rubble of various sizes. Therefore, an MRR must have a mobility system capable of traversing such terrain, surmounting rubble, and even climbing steps. Additionally, the size and weight of a robot increase with the sophistication of the incorporation of functionalities and requirements into the robot. Advanced mapping capabilities increase weight and size because they require a versatile sensor suite, microcontrollers with sufficient computational power, and sufficient energy sources. The permissibility requirements for intrinsically safe, explosion-proof, and waterproof designs also affect the weight because they require specific materials, capsules, and partitions for electric component isolation, etc. Therefore, the principal challenge in terms of size and weight of a robot is the efficient combination of all the design element requirements. Table 4 summarizes the known weights and dimensions of the reviewed MRRs (without considering the most lightweight surveying and mapping platforms, Souryu series platforms, and Subterranean Robot). The heaviest platform is the Mobile Inspection Platform, whereas the lightest platforms are Gemini-Scout and MINBOT-II. The remaining platforms weigh between 450 kg and 590 kg.
2. Intrinsic safety and explosion-proof design: In underground mines, especially coal mines, combustible gases, dust, and vapors are present in greater quantities than in standard air and can potentially be ignited with fire, electronic sparks, or even electrostatic sparks, leading to a coal mine disaster (Rong et al., 2011).

Intrinsic safety refers to limiting electrical energy transferred between circuits to nonhazardous levels in order to prevent the device from producing a spark that can ignite combustible atmospheres. This is achieved using appropriate electrical design, intrinsic barriers, and antistatic components that are certified to make the device incapable of producing a spark. Explosion-proof design aims to contain and survive an explosion, preventing it from spreading outside the equipment. This is achieved mainly through the mechanical design of explosion-proof housings and compartments that enclose electrical devices. In general, intrinsically safe equipment operates at lower energy levels than explosion-proof equipment, reducing the thermal and electrical energy released and preventing explosions from happening in the first place. Explosion-proof equipment operates at normal energy levels and is designed to prevent heat and flames from reaching hazardous levels that could cause an explosion.

**TABLE 4:** Weight and dimensions of selected reviewed MRRs.

CMRR	Weight (kg)	Dimension (mm) W × L × H
Numbat (Ralston et al., 2001)	(n.d)	1,650 × 2,500 (n.d)
ANDROS Wolverine (Murphy et al., 2009; Li et al., 2020)	544	760 (n.d) × 1,270
Gemini-Scout (Reddy et al., 2015; Salton et al., 2017; Callow, 2018)	86	600 × 1,200 × 600
MINBOT-II (Reddy et al., 2015; Salton et al., 2017; Callow, 2018)	130	589 × 1,500 × 747 (with arms extended)
CUMT-V (Zhao et al., 2017; Li et al., 2022)	430	536 × 1,315 × 862
MPI (Novák et al., 2018; Bołoz and Biały, 2020; Kasprzyczak et al., 2016)	1100	1,150 × 2,400 × 1,800
TeleRescuer (Kasprzyczak et al., 2016; Moczulski et al., 2014; Kot et al., 2017)	590	741 × 2,100 × 500–920

Although intrinsically safe devices are lighter in weight and offer more flexibility in usage, they are not as readily available in the market. As a result, robot designers commonly use existing commercial devices enclosed in explosion-proof containers. As discussed in Section 5.1.2, many countries have established regulations that define the type and procedures for using electrical and electronic equipment in potentially flammable and explosive atmospheres (Calder et al., 2018). Flameproof enclosures and pressured enclosures are two of the most common types of explosion-proof enclosures. Ideally, flameproof enclosures should possess sufficient mechanical strength to withstand the impact of a potential explosion within them, as well as prevent a fire or electronic spark from starting the explosion outside. In pressured enclosures, a gas supply device inside keeps the enclosure airtight. The mechanical tensile strength of this enclosure is much lower than that of the flameproof enclosure. In this respect, the pressured enclosure is much lighter than the flameproof enclosure with the same volume of interior space. An “explosion-protection” technique is used to reduce the possibility that electrical and electronic equipment may ignite in a hazardous location, termed HazLoc. Explosion protection for equipment can be achieved in three basic ways: preventing the gas from reaching the circuitry, limiting the circuitry’s energy (to less than 0.3 mJ) under normal operating conditions and two simultaneous faults, so that ignition is not possible, or containing the explosion to the immediate area surrounding the circuitry to prevent it from spreading (Calder et al., 2018).

Most of the reviewed MRRs are explosion proof because their objective is to be used in mine emergencies. The detailed documentation provided by the developers of the MINBOT-II (Wang et al., 2014), CUMT-V (Li et al., 2020), MPI (Kasprzyczak et al., 2016), and TeleRescuer (Novák et al., 2018) offers an insightful overview of intrinsically safe and explosion-proof techniques. Notably, the Gemini-Scout is NIOSH permissible to be used in mines, the CUMT-V platform has obtained the Mining Products Safety Approval and Certification (China), while the MPI and TeleRescuer platforms are developed according to the ATEX (EUR-Lex, 2014) and IEC 60079 standards (IEC, 2017), respectively.

1. Waterproof design: It is apparent that a waterproof design for all the electrical components of a disaster robot is essential for the robot to be able to operate. The Australian pipeline robot deployed at the Peck River Coal Mine disaster failed due to a short circuit that occurred after the robot walked only 500 m (McDonald, 2023). Considering the reviewed MRRs regarding waterproof design: Numbat has its electronic components sealed and also uses nitrogen-filled compartments (Murphy et al., 2009); Gemini-Scout can function in water and wet rock dust up to 45 cm deep (Deuel et al., 2018); the Subterranean Robot is amphibious by design and can operate underwater up to 10 m (Maity et al., 2013; Ray et al., 2009); Souryu-V design includes pressure-resistant seals for waterproofing electrical components (Arai et al., 2008); the MINBOT-II and CUMT-V design includes both whole-body static sealing and a power output shaft dynamic sealing design, achieving thus IP67 protection (immersion in water up to 1 m for 30 min) (Zhao et al., 2017; Wang et al., 2014). ANDROS Wolverine, MPI, and TeleRescuer have not reported waterproofing design, while the surveying and mapping robots Groundhog and Cave Crawler are reported as not waterproof platforms.

#### 4.2.8 Firefighting capabilities

Firefighting capabilities are another topic of interest in the context of requirements for mine search and rescue robots. Fires are a common occurrence in a post-disaster mine environment, and the ability for a robot to put out small fires on its pathways would increase its operational range and potential in mine search and rescue operations. The designers of an MRR intended for firefighting must consider (at least): a) the mechanical elements that store and deploy fire extinguishing foams, b) high-temperature resistant design of the robot, and c) sensor suites and software stack that can identify fires and navigate in smoke. Deuel et al. (2018) explore common design considerations for the protection of robots exposed in hazardous conditions, such as potentially explosive atmospheres, firefighting operations, biochemical sampling, and radiation, and discuss examples of firefighting robots. Considering the reviewed MRRs, only CUMT-V is equipped with a small amount of dry powder fire extinguisher (Li et al., 2020).

#### 4.2.9 Human–robot interaction (HRI)

Disaster robotics requires the integration of intuitive interfaces, communication modalities, and ergonomic designs that allow a remote human user to seamlessly supervise and operate the robot. A remote operator station combining a control device (typically a joystick), a video feed, sensor data displays, and an emergency stop is a baseline requirement for every reviewed MRR.

Human–robot interaction (HRI) is a complex component of a robotic platform due to the multi-disciplinary elements involved in its design. Effective and intuitive control of all the operational, mechanical, and software-related capabilities of the robot is one of the principal objectives of an HRI framework. However, in addition to functionality control, HRI must encompass selection and development of both hardware and software components capable not only of visualizing the data collected by the robot’s sensor suite, but also of processing, interpreting, and extracting salient features from the data. Additionally, the HRI design must effectively present the extracted features to the human operator in a manner that improves the operator’s situational awareness and reaction time. A wide variety of tools is available, spanning from visual displays to speech and auditory signals, haptic and gesture-based interfaces to virtual reality headsets, and even anthropomorphism and proxemics. HRI design and development must also consider human factors and appropriate training to prepare a human operator to collaborate with a robot. This means that aspects of situational perception, psychomotor aspects, attentional control, perceptional overstimulation, working memory capacity, fatigue, trust, and complacency are involved in the design.

Although the available information for the reviewed MRRs does not provide details on their respective HRI components (except for a few images depicting aspects of the GUIs (Li et al., 2020; Wang et al., 2014; Kasprzyczak et al., 2016)), the vitality of an efficient HRI has been emphasized. Murphy et al. (2009) describes the failed rescue deployment of ANDROS Wolverine at the Sago Mine disaster (January 2006) when the teleoperated robot ran off its path in a dip due to the lack of adequate situational awareness provided by the HRI to the operator. The same study points out that “without good HRI, the robot may roll past signs of survivors or imminent hazards,” while the delineated requirements for an HRI include the ability to minimize the number of humans needed and the inclusion of both proprioceptive and exproprioceptive data displays. Fundamental exproprioceptive data, as Deuel et al. (2018) suggest, include the addition of robot health status monitoring at the user interface of the Gemini-Scout robot as a future goal.

#### 4.2.10 Redundancy

A disaster robotic platform runs the risk of losing sensors and functionalities or being immobilized or destroyed while exercising a mission. This can be the result of the extreme conditions of a post-disaster environment, human operator error, or failure of poorly designed mechanical or software elements. Therefore, a mine rescue robotic platform must be designed for redundancy. Ideally, redundancy designs would involve all the previously discussed aspects of robot design: electric equipment malfunctions, power sources, mobility and manipulation, sensors, and communication. However, the increased complexity of a holistically redundant robot would significantly increase the size and weight of the platform, if it even allowed the development of such a platform. Nevertheless, subsystems of a robotic platform, especially those more fault-susceptible, should integrate redundancy designs. Fail-safe mechanisms and fault-tolerant design for the sensor suite, the communication components, and even the core processors are commonly applied in exploration robots through techniques such as duplicate or overlapping components, regular backups, and automatic troubleshooting and processor restarting. Safeguarding and redundancy design in disaster robotics are extremely vital for preventing the loss of collected data, even in cases when the robot is destroyed or immobilized.

The investigated MRRs, although not directly focusing on redundancy, are equipped with multiple cameras that duplicate or overlap, and on safeguarding electronic devices. In addition to the reviewed MRRs, a commonly used technique in recently developed unmanned subterranean exploration systems is the deployment of multi-agent collaborative robotic systems. Example cases were shown during the DARPA Subterranean Challenge (DARPA, 2022). These designs consist of a central operation station that commands two or more unmanned robotic agents, mainly UGVs and UAVs, that have different capabilities and are assigned different tasks to ensure the seamless operation of the whole system (Agha et al., 2021; Hudson et al., 2021; Roucek et al., 2021; Tranzatto et al., 2022). These multi-agent systems often combine agents that are exact duplicates and are deployed for the same tasks either in a serial manner or as replacements for a failed agent, for example, teams MARBLE and Explorer (Ackerman, 2023). These strategies offer great flexibility, both in development and deployment, and a redundancy level that can even eliminate the single-point failure problem.

## 5 Discussion

This review examines the mechanical and hardware aspects of 15 documented coal mine rescue robots developed over the last 3 decades. The focus is on the design requirements and trade-offs that govern the mechanical and computational architecture of individual platforms, with the aim of supporting future development of robotic systems that can effectively be deployed in a harsh post-disaster mining environment. Existing reviews on the subject are either not comprehensive regarding the design requirements and specifications or are no longer current. The present work offers an updated discussion of CMRRs that combines and extends these earlier efforts. The discussion below examines lessons learned, current design standards, and common design elements from the standpoint of stand-alone robotic agents, followed by emerging research directions relevant to coal mine rescue applications.

### 5.1 Key design requirements

The recent technological advances in fields such as robotics and computational architectures and software development, sensors, and communications have led to the development of increasingly sophisticated disaster robotics. The modern MRRs are characterized by increased sophistication in mechanical and software design, while simultaneously emphasizing alignment with permissibility and safety regulations and standards. Naturally, these MRRs have significantly increased size and weight. Most of these MRRs hold a permissibility classification and certification, such as ATEX (Europe), MA Certification (China), NIOSH permissibility certification (United States), and IP67 (waterproof) protection standard (international). However, none of these robots have been deployed in an active search and rescue mission. This phenomenon can be attributed to the significant reduction of mining disasters through the revision and stricter enforcement of health and safety regulations in recent years. However, it should be noted that the same regulations have made the certification and thus testing and deployment of such MRRs in mines, whether for routine or emergency operations, harder to acquire.

Despite the advances in robotics, fully autonomous disaster robotics are not yet capable of operating seamlessly in disaster environments. However, human-operated robotic platforms have been proven valuable in reconnaissance of post-disaster underground mines. As Murphy et al. (2009) stated, “Although mine robots have not found any survivors to date, they have already proven to be a valuable asset for mine rescue teams by providing video and atmospheric monitoring information at several emergency and recovery sites.” The same study points out two emergency cases where rescue teams were provided with recordings of the mine atmosphere conditions from a deployed robot. The rescuers were better prepared to enter the mine or avoided risking their lives by entering an extremely toxic mine site. This observation makes apparent the usefulness and vitality of disaster robotics in mine emergencies, even if the current technology does not guarantee the survivability of an individual robotic agent.

Moreover, it is important to acknowledge that the limited practical deployment of CMRRs stems not solely from technical shortcomings of individual platforms, but from the inherent complexity of mine search and rescue as a systemic challenge. Effective rescue operations require seamless integration across multiple domains: accurate surface and subsurface detection for locating trapped miners, specialized mechatronics components for access in challenging terrains, coordinated multi-agent systems, sophisticated management frameworks for resource allocation, command and control, and adaptive rescue tactics. Recent developments in collaborative robotics, exemplified by the DARPA Subterranean Challenge (RoboCup Federation, 2023), demonstrate that teams of diverse robotic agents (aerial, ground, wheeled, or legged) can successfully explore complex underground environments through coordinated action. Furthermore, advances in self-supervised learning enable robots to rapidly adapt to novel terrains and failure modes, suggesting promising paths forward for disaster robotics. While this review focuses on individual CMRR platforms and their design requirements, these broader systemic considerations remain essential for successful real-world deployment.

The discussion below regarding the reviewed MRRs will attempt to pinpoint lessons learned, current approaches to design standards and design elements from the standpoint of stand-alone robotic agents, and future trends.

#### 5.1.1 Key design elements

**FIGURE 4:**
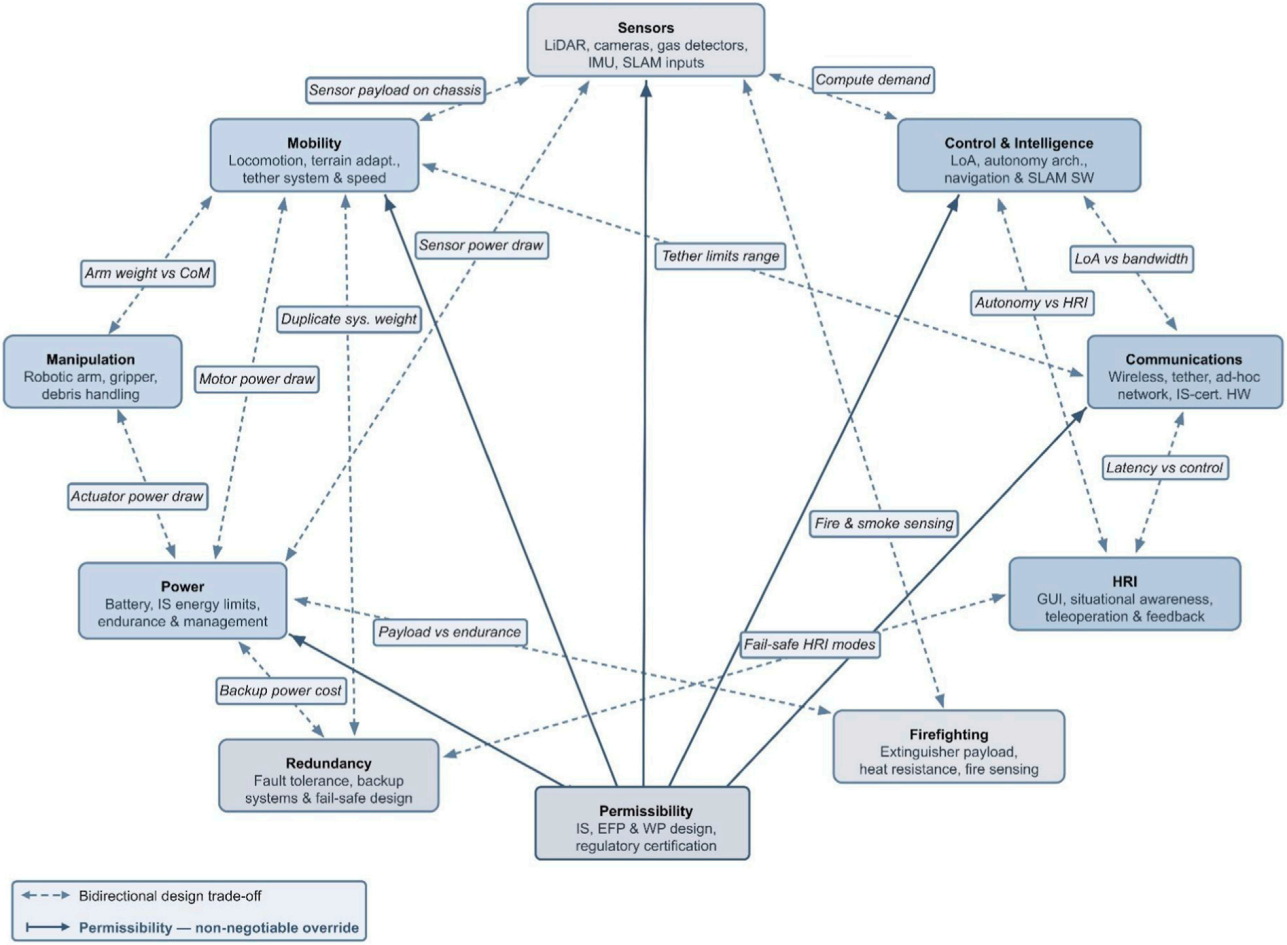
Diagram of inter-element design trade-offs and constraints for CMRRs. (Abbreviations: IS, intrinsically safe; EFP, explosion- and fire-proof; WP, waterproof; HW, hardware; SW, software; LoA, level of autonomy; CoM, center of mass).

**FIGURE 5:**
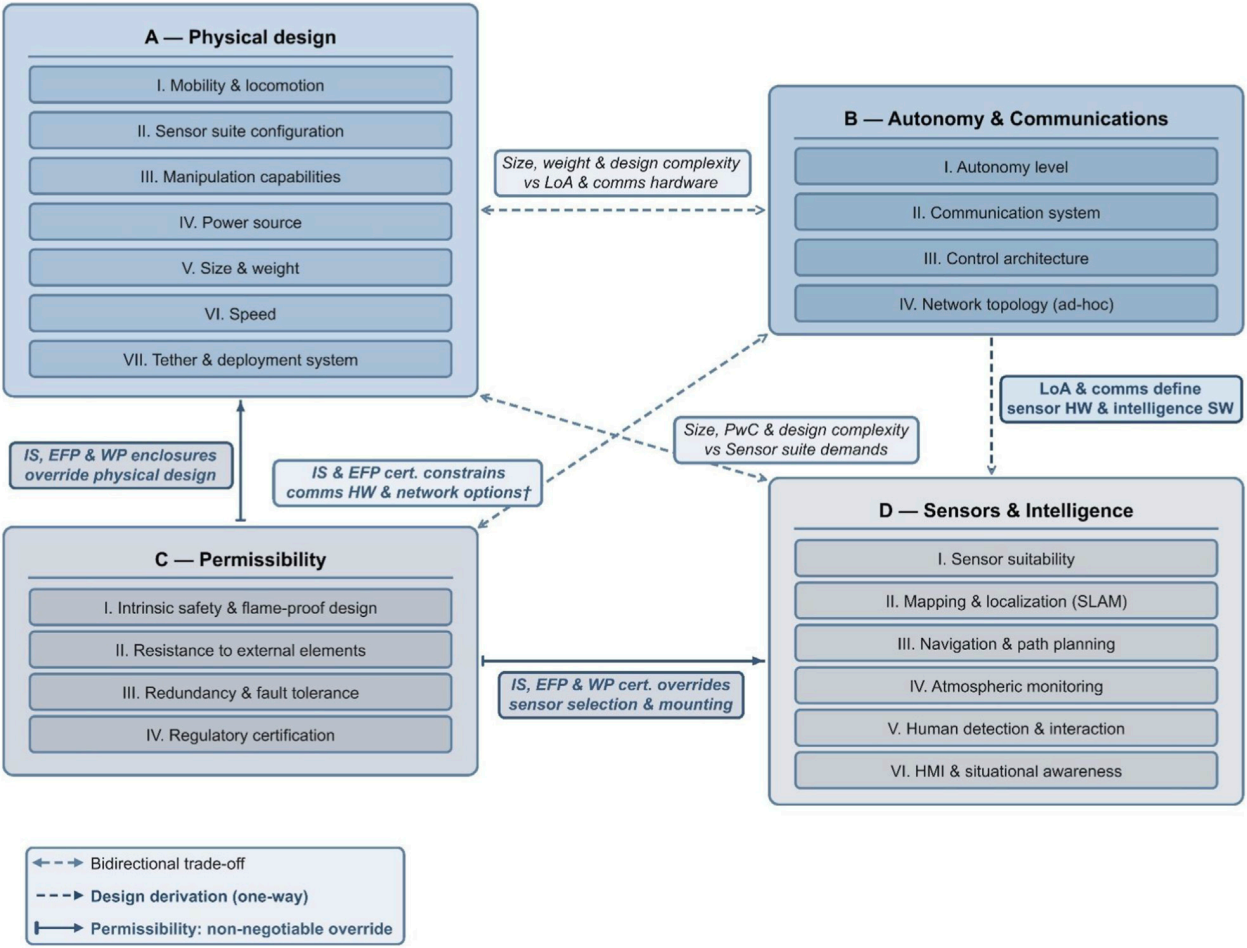
Diagram of inter-category design trade-offs and constraints for CMRRs. (Abbreviations: LoA, level of autonomy; PwC, power consumption; IS, intrinsically safe; EFP, explosion- and fire-proof; WP, waterproof; HW, hardware; SW, software).

The design considerations can be divided into four categories: (A) physical design, (B) autonomy and communications, (C) permissibility, and (D) sensors and intelligence. These form a structured network of mutual trade-offs, derivation relationships, and regulatory constraints, as illustrated in Figures 4, 5. In general, categories B and C jointly occupy a position of priority: B defines the operational ambition, C filters the implementable solution space, while A and D are the optimized physical solutions. Detailed inter-category coupling is discussed in Section 5.1.3. The common design considerations for deployment in mine rescue operations can be summarized as follows:

##### 5.1.1.1 Physical design

- Mobility: The locomotion system must enable the platform to traverse harsh terrain and surmount rubble and steps. The platform must handle and remain functional on off-road, highly uneven, inclined, slippery, debris-filled, and dusty terrain. A combination of wheels, tracks, and tracked arms has provided the most adaptable approaches based on the reviewed MRRs.
- Sensor suite mechanical configuration: The sensors must be integrated properly on the platform so that unobstructed data are collected while the sensor suite configuration minimizes potential tear-and-wear through entanglement or contact with walls, roofs, hanging, or protruding elements. The sensors mounted on the platform must mitigate external disturbances, for example, magnetic interference, vibrations, and rotations. Potential solutions are proper placement of sensors, absorbing mediums, suspension systems, and gimbals. Additionally, the sensors must be capable of self-cleaning.
- Manipulation capabilities: The integration of dexterous arms and grippers that can handle switches, fans, or tools, open doors, move small objects, obstacles, or rubble is a highly desirable design feature, but the current state of the art is not appealing to the MRR developers (based on the examination of the reviewed MRRs).
- Power source: An onboard power source is an indispensable necessity for every modern robot. Considering the subterranean environments and the potentially explosive atmospheres, the only option is electric batteries. Intensive research in this field has yielded batteries capable of supporting MRRs for several hours.
- Size and weight: Maneuverability, access to confined spaces, and transportation/portability are factors significantly influenced by the size and weight of the robot. Size and weight are defined by the compound effect of all the other design elements and practices.

##### 5.1.1.2 Autonomy level and communications

- Autonomy level: The autonomy level of an MRR falls into a spectrum between remote operation (teleoperation) and fully autonomous operation. Although teleoperated MRRs have been successfully deployed, the acquisition of seamless situational awareness from the operator is challenging (e.g., ANDROS Wolverine failure in the Sago Mine deployment).
- As noted by Murphy et al. (2009), “a single operator could not keep up with where the robot was and keep on looking.” On the other side, fully autonomous disaster robotics are not yet technologically mature. Therefore, a system that retains a human in the loop is the common approach of modern MRRs. A combination of teleoperation, semi-autonomous, and fully autonomous features and capabilities combines the benefits of each approach and allows for flexibility.
- Communications: A robotic platform can utilize wireless technologies or cables for control commands and data communication, while some robots integrate both approaches. Wired and fiber-optic channels offer important advantages in coal mine conditions: immunity to the severe RF attenuation caused by coal seams and wet rock, deterministic low-latency links capable of supporting high-definition video and high-rate LiDAR streaming, and a substantially simpler path to ATEX/MA certification compared with active wireless transmitters operating in Zone 0 explosive atmospheres. These properties make a wired tether the pragmatic preferred choice for linear-drift reconnaissance from the fresh air base and for platforms that must relay continuous high-bandwidth sensor data. However, tether range and the risk of entanglement or breakage impose operational constraints that wireless approaches overcome. Murphy et al. (Murphy et al., 2009), based on the deployments of ANDROS Wolverine, reported that “the use of fiber-optic cables for communications between the robot and operator is not viable even for short distances, and wireless solutions are needed.” The optimal architecture, as demonstrated by CUMT-V and TeleRescuer, combines fiber-optic reliability with wireless redundancy, treating the two as complementary. Platforms that can independently create *ad hoc* wireless networks through intelligent deployment of communication nodes are a topic of intensive research.

##### 5.1.1.3 Permissible electronics

- Intrinsic safety, explosion-proof, and flameproof design: The subterranean mine atmosphere and environment must be protected by all the electric and structural components of the platform. Intrinsically safe electronics and antistatic components minimize the potential of creating sparks and electric arcs, while explosion-proof and flameproof enclosures can contain a potential fire caused by robots. The detailed international and regional standards extensively regulate the appropriate methods and techniques to achieve this type of protection.
- Resistance to external elements: The platform’s components must be protected from water, humidity, high temperature, minor rockfalls, corrosive substances, and radioactive material. Solutions can be provided by advanced materials and alloys that are extremely damage-resistant and lightweight.
- Redundancy: Redundancy must be integrated for all the core physical and intelligence components. Common practices include fail-safe mechanisms, fault-tolerant designs for the sensor suite, the communication components, and the core processors, regular backups, automatic troubleshooting, and processor restarting.

##### 5.1.1.4 Sensors and intelligence

- Sensor suitability: The selected sensor suite must be selected according to the desired intelligence capabilities. Accuracy and resolution of sensors must meet the minimum requirements for the respective intelligent functionalities.
- Mapping and localization: A disaster robot must be capable of creating at least 2.5D maps of GPS-denied environments and localize itself with regard to static and dynamic obstacles. Advanced algorithms, such as SLAM, are commonly applied.
- Navigation and path planning: A robot must be able to recognize terrain conditions, static and dynamic obstacles, crevices, rubble piles, hanging or protruding objects, and appropriately plan the safest paths to avoid or surmount obstacles.
- Atmospheric conditions recording: A priority capability for MRRs is to relay information about the atmosphere of the mine to the rescue teams and the command center. Gas sensors in combination with mapping can create 2D maps that are easily readable by humans.
- Recognize and fight fires: Although not a capability considered by most of the reviewed MRRs (only CUMT-V has firefighting ability), it is a desirable capability.
- Recognize and interact with humans: Recognize humans as well as their consciousness and physical state. Two-way voice capabilities are also a fundamental functionality of most of the reviewed MRRs.
- Human–robot interaction: A critical aspect of any MRR is the development of intuitive and informative HRI protocols and GUIs that enable efficient situational awareness and control to a remote user. Additionally, interaction frameworks that allow for a human operator to collaborate and interact with a robot while walking alongside or at a safe distance from the robot are often needed.

The lack of widely accepted standards leads MRR developers to focus on different sets of design elements. Naturally, it follows that success and failure factors differ between platforms. As an attempt to summarize the focus of the MRR developers, Table 5 lists the most discussed design aspects reported by the developers. Additionally, the successful and challenging design elements of the reviewed MRRs are summarized and categorized based on the common design considerations specified in Table 6.

**TABLE 5:** Critical design elements of MRRs based on focus of technical disseminations.

Robotic platform	Key design element	Category
RATLER (Krotkov et al., 1994; Klarer, 1993; Purvis and Klarer, 1994)	Mobility in lunar-like terrain Power source	A.I A.IV
Numbat (Ralston et al., 2001; Hainsworth, 2001; Ralston and Hainsworth, 1998)	Mobility in harsh terrain Remote operation/comms Comms cable release system (12 km range) Explosion-proof and waterproof design Real-time GUI	A.I B.I, B.II B.II C.I, C.II D.VII
ANDROS Wolverine/V2 (Murphy et al., 2009)	Mobility in harsh terrain Comms cable (1.5 km range) Explosion-proof and waterproof design	A.I B.II C.I, C.II
Groundhog and Cave Crawler (Masayuki et al., 2004; Casper et al., 2004; Murphy, 2010)	Mobility in harsh terrain Lightweight design Advanced mapping and navigation	A.I A.V D.I, D.II, D.III
Souryu series (Arai et al., 2008; Masayuki et al., 2004)	Mobility mechanisms for rubble Lightweight design Water- and dust-proof design	A.I A.V C.II
Inuktun VGTV (Murphy et al., 2009; Murphy, 2010)	High traction mechanism	A.I
WA Water Service Company Robot	*Insufficient information*	-
Subterranean Robot (Reddy et al., 2015; Ray et al., 2009; Maity et al., 2013)	Hybrid mobility Wireless RF/WLAN comms Waterproof design Obstacle avoidance and navigation Real-time GUI	A.I B.II C.II C.V D.VII
Leader	*Insufficient information*	-
Gemini-Scout (Green, 2013; Reddy et al., 2015; Deuel et al., 2018)	Mobility in harsh terrain Sporadic steering commands Remote operation/comms Intrinsically safe design (C.I)	A.I B.I B.I, B.II C.I
MINBOT-II (Zhao et al., 2017; Wang et al., 2014)	Mobility and locomotion system Sensor positioning Power supply Autonomy level and control Communications Explosion-proof and waterproof design Path planning software module	A.I A.III, D.I A.IV B.I, D.VII B.II C.I, C.II D.III
CUMT-V (Li et al., 2020)	Mobility and locomotion system Sensor positioning and perception Autonomy switching and control performance Communications and cable relay system Multi-robot system for increased operation range Intrinsically safe design	A.I A.III, D.I B.I, D.VII B.II B.II C.I, C.II
Mobile Inspection Platform (Novák et al., 2018; Bołoz and Biały, 2020; Kasprzyczak et al., 2016)	Intrinsically safe design Sensor positioning and perception Communications system	C.I, C.II A.III, D.I, D.IV B.II
TeleRescuer (Novák et al., 2018; Cyran et al., 2018; Moczulski et al., 2014; Kot et al., 2017; Kot et al., 2016)	Mobility and locomotion system Sensor positioning and perception Communications and cable relay system Intrinsically safe design 3D LiDAR-based mapping Control and GUI	A.I A.III, D.I B.I, B. II C.I, C.II D.II D.VII

**TABLE 6:** Success and failure design factors of reviewed MRRs.

Robotic platform	Success factor	Challenging or failure factors
RATLER (Purvis and Klarer, 1994; Ralston et al., 2001; Hainsworth, 2001)	Mobility in lunar-like environments (A.I)	Limited maneuverability (A.I, A.V) (Li et al., 2020) Communication range <76 m (B.II) (Li et al., 2020; Wang et al., 2018)
Numbat (Ralston et al., 2001; Hainsworth, 2001; Ralston and Hainsworth, 1998)	Mobility in harsh terrain (A.I) Remote operation/Comms (B.I, B.II) Explosion-proof and waterproof design (C.I, C.II) Real-time control and GUI (D.VII)	Large dimensions restrict access to mines (A.IV) (Molyneaux et al., 2015)
ANDROS Wolverine (Murphy et al., 2009)	Mobility in harsh terrain (A.I) Able to move debris, close doors (A.II) Remote operation/comms (B.I, B.II) Mine-permissible design (C.I, C.II) Able to collect gas samples (D.IV)	Height clearance issues (A.IV) (Murphy et al., 2009) Fiber-optic cable breakage (B.II) (Murphy et al., 2009) Blind spots for remote operator (D.VII) (Murphy et al., 2009)
Groundhog and Cave Crawler (Masayuki et al., 2004; Casper et al., 2004; Murphy, 2010)	Mobility and portability (A.I, A.V) Fully autonomous robots (B.I) Adaptable mapping system (C.II) Effective mine navigation (C.III)	Not an MRR Not mine-permissible
Souryu series (Arai et al., 2008; Masayuki et al., 2004)	Mobility in harsh environments (A.I) Sensor positioning flexibility (A.III) Size, maneuverability, portability (A.V) Water- and dust-proof mechanism (C.II)	Challenges with cascading or angled obstacles (A.I) (Reuters, 2010) Blind spots for remote operator (D.VII) (Reuters, 2010)
Inuktun VGTV (Murphy et al., 2009; Murphy, 2010)	Vertical mobility (A.I) Portability for deployment (B.IV)	Significant navigational challenges (A.I) (Murphy, 2010) Limited visibility of cameras w/o lights (A.III) (Murphy et al., 2009) Challenging to keep camera lenses clean (A.III) (Murphy et al., 2009)
WA Water Service Company Robot	*Insufficient information*	*Insufficient information*
Subterranean Robot (Ray et al., 2009; Maity et al., 2013)	Hybrid mobility (A.I) Wireless RF/WLAN comms (B.II) Waterproof design (C.II) Sufficient obstacle detection sonar (D.I) Real-time control and GUI (D.VII)	Floor slippage (A.I, D.III) (Ray et al., 2009; Maity et al., 2013) Path deviation due to data error (D.I, D.III) (Maity et al., 2013)
Leader	*Insufficient information*	*Insufficient information*
Gemini-Scout (EUR-Lex, 2014; Kasprzyczak et al., 2016; Cyran et al., 2018; Moczulski et al., 2014)	Mobility over boulders and rubble (A.I) Lightweight design (A.V) Remote operation/comms (B.I, B.II) Mine-permissible design (C.I, C.II) Intuitive control system (D.VII)	Not fully submersible, only up to 450 mm (C.II) (Molyneaux et al., 2015) *(insufficient information on performance testing and challenges)*
MINBOT-II (Zhao et al., 2017; Wang et al., 2014)	Mobility over slopes, rubble, steps (A.I) Multiple powering options (A.IV) Multiple autonomy levels (B.I) Multiple comms modes (B.II) Explosion-proof and waterproof design (C.I, C.II)	Difficulties driving over rails (A.I) (Zhao et al., 2017) Quick voltage loss with cable power supply (A.IV) (Zhao et al., 2017) Unstable wireless comms (B.II) (Zhao et al., 2017) Fiber-optic cable unfolding problems (B.II) (Zhao et al., 2017) Cable weight limits agility (A.IV, B.II) (Zhao et al., 2017) Video and audio affected by vibrations (A.III, D.I) (Zhao et al., 2017)
CUMT-V (Li et al., 2020)	Mobility tests show high terrain adaptability (A.I) Multiple autonomy levels (B.I) Mine-permissible design (C.I, C.II) Extensive sensor suite (D.I) Firefighting capability (D.V) Real-time control and GUI (D.VII)	Track-separation incidents (A.I) (Li et al., 2020) Rear-movement braking problems (A.I) (Li et al., 2020) Camera glare problem (A.III, C.I) (Li et al., 2020) Fiber-optic cable unfolding problems (B.II) (Li et al., 2020)
Mobile Inspection Platform (Novák et al., 2018; Bołoz and Biały, 2020; Kasprzyczak et al., 2016)	Successful mobility/traction tests (A.I) Reliable wireless system function (B.II) Intrinsically safe design (C.I, C.II) Extensive sensor suite (D.I) Video feed in poorly lit spaces (A.III, D.I) Real-time control and GUI (D.VII)	Limited flexibility due to size and weight (A.V) (Novák et al., 2018) Limited autonomy (B.I) (Kasprzyczak et al., 2016) *(insufficient information on performance testing and challenges)*
TeleRescuer (Novák et al., 2018; Cyran et al., 2018; Moczulski et al., 2014; Kot et al., 2017; Kot et al., 2016)	Reliable wireless system function (B.II) Intrinsically safe design (C.I, C.II) Hardware and software fail-safe systems (C.III) Extensive sensor suite (D.I) Fast point-cloud scanning (D.II) Real-time control/GUI (D.VII)	*Insufficient information on performance testing and challenges*

The design aspects that have received attention in all the reviewed MRRs are i) locomotion that allows flexible and reliable mobility on harsh terrain (A.I), ii) robust communications system, whether wireless or cable-based (B.II), iii) explosion-, flame-, and waterproofing (C.I and C.II), and iv) sensor suite selection for atmospheric conditions monitoring (D.IV). In more recently developed MRRs, the developers also report on autonomy level (B.I) in conjunction with control system and human–machine interface design (D.VII). Mapping (D.II) or path planning and autonomous navigation considerations (D.III) are usually briefly discussed or omitted. However, this can be attributed to the fact that many MRRs are designed as teleoperated or dual autonomy modes, and therefore the focus of performance testing with regard to sensory input and path planning focuses mostly on the clarity and consistent functionality of visual sensors.

On the other hand, the most challenging or failure aspects reported include:

- Limitations or specific failure incidents of the locomotion system, such as track separation, challenging reverse movement, challenges or failure to traverse a specific configuration of obstacles (category A.I of requirements),
- Challenges and noise interference with sensory input such as noise due to vibrations, impact sounds overlapping audio, camera glare because of transparent enclosures, limited visibility because of mud, dust or poor illumination (A.III),
- Limitations in flexibility and traversability of narrow spaces due to size and weight (A.V).
- Stability and range of communications (B.I and B.II),
- Tether breakage, entanglement, or maneuverability limitations caused by the cable weight or relay system (B.II),
- Challenges in teleoperation due to insufficient information relayed or presentation manner to the handler (C.III, D.I, and D.VII), and
- Challenges in autonomous operation, mapping, and path planning (B.I, D.II, and D.III).

#### 5.1.2 Design standards

The established design standards (described in Section 4.1 and listed in Supplementary Appendix Table A1) provide strict guidelines for electric equipment specifications that are relevant to the design of safe and efficient robots for deployment in outdoor environments. These guidelines are imperative for ensuring that the different electrical components of a robot can operate in harsh outdoor environments (e.g., waterproof and resistance to high heat) while also not acting as potential sources of hazards for this environment (e.g., explosion proof and intrinsically safe equipment). The standards can be roughly divided into three categories: i) equipment protection through segregation, ii) explosion or fire containment, and iii) elimination or mitigation of hazard creation through preventive measures. The most common approaches dictated in the standards pertain to:

- Sealing and enclosure of electrical equipment: Sensors and electrical components, including their connections, conductors, and batteries, are sealed for protection. Flameproof, explosion-proof, and waterproof enclosures that are made so through pressurized enclosures, powder filling, immersion in liquid, and other techniques are described.
- Intrinsically safe electrical equipment: Standards dictate specifications of intrinsically safe devices regarding surface heat, radiation emission, sparks, etc.
- Detection of hazards: Gas, dust, temperature, and radiation detectors are described in the standards as tools for hazard detection. In the scope of disaster robotics, these detectors can be utilized both for data collection and as emergency shut-off triggers.

The most followed approach of the reviewed MRRs is the sealing and enclosure of electrical components. Although these standards ensure safe integration and implementation of electronic equipment, they do so on a small scale by tackling different components separately. In the scope of developing a robotic platform comprised of multiple different electrical components that must be placed at different points on the platform or even exposed to the open air, the standards can introduce heavy complexities and even limit the capabilities of the platforms. It has been demonstrated that attempting adherence to such standards leads to big and heavy MRRs that may have limited maneuverability or ability to penetrate tight spaces (e.g., NUMBAT, MPI, and TeleRescuer). This stems from the need to integrate housing and compartments for various mechanical components and sensors. Notably, the approach that increases platform weight is the integration of explosion containment enclosures that must be made of sturdy enough materials that can contain high temperatures (at least 150 °C) and high internal explosive pressure (at least 10.34 bar or 150 psi). Additional challenges have to do with obstructions or interference to the sensors’ ability to collect data due to the enclosures’ material or configuration. However, the continuous advances in material fabrication and the design of commercially available intrinsically safe sensors have already been facilitating and mitigating these challenges.

#### 5.1.3 Interdependencies among design element categories

The interdependencies between the design categories can be explained through the following four points:

1. Priority of B and C over A and D. Autonomy and communications architecture (B) establishes the operational ambition of the robot: the required LoA, communications range and bandwidth, control architecture, and network topology. These parameters collectively determine what the physical platform (A) must carry and what the sensor and intelligence suite (D) must deliver. A robot targeting high LoA requires a different chassis geometry, power budget, and locomotion system than a tethered teleoperation platform. A-type requirements (mobility, mounting, size, weight, speed, tether/no tether) are a downstream consequence of B rather than an independent design choice. D is likewise derived from B: the sensor suite, SLAM capability, and onboard intelligence required are defined by the autonomy level and communications architecture. This derivation, denoted B→D in Figure 4, reflects a design sequence rather than a symmetric trade-off.
2. A and D as mutual trade-offs within B-defined bounds. A and D are not independent of each other. A larger sensor suite increases the size, weight, and power consumption of A, while the physical geometry and mobility constraints of A limit what sensor configurations are mountable and operable. This mutual trade-off (A↔D in Figure 4) must be co-optimized within the envelope established by B and filtered by C. A and B similarly engage in a moderate bidirectional trade-off: chassis dimensions, locomotion type, and tether architecture constrain achievable communications range, while higher LoA and more sophisticated communications hardware impose additional size and power demands on A.
3. C is an engineering vocabulary. Permissibility requirements (C) can be viewed as the engineering vocabulary from which designers select certified solutions to implement B in A and D. IS-certified processors, explosion-proof (EFP)-rated enclosure manufacturers, and ATEX-approved wireless modules are commercially available products. The practical development workflow is to define the autonomy and communications ambitions of B first, then identify which certified components within C can deliver the physical and sensing implementations required by A and D. The constraints that C imposes on A and D, meaning IS and EFP-rated enclosures, waterproofing and dust resistance for sensor mounting, regulatory compliance of all electronics, are the filters on the solution space of A and D.
4. B↔C confrontation. The other side of point 3 is that permissibility requirements directly constrain autonomy ambitions. This reframing does not hold for the software-defined sub-elements of B, that is, B.I (LoA as an algorithmic capability) and B.III (control architecture as a software framework). These sub-elements are not directly subject to IS or EFP certification and retain full design freedom. However, the hardware-facing sub-elements, that is, B.II (communications system) and B.IV (network topology), engage in a direct confrontation with C. IS-certified wireless hardware operates at lower transmission power than non-certified equivalents, imposing hard limits on bandwidth, range, and latency. Because higher LoA demands higher-bandwidth sensor streaming and lower-latency control, developers targeting high LoA may find that no IS-certified wireless solution can support the required data throughput, forcing a genuine scaling back of B.I rather than a substitution in A or D. IS certification similarly limits electrical energy storage and dissipation, constraining onboard computer power because IS-certified embedded processors are significantly less powerful than non-certified equivalents, representing a direct ceiling on B.I not solvable through choices in A or D. Battery energy density is subject to the same constraint, capping mission endurance as a B-level operational parameter. The B↔C relationship is therefore asymmetric and sub-element specific. This is reflected in the moderate coupling strength of the C × B cell in Figure 5, contrasting with the strong overrides of C × A and C × D.

In summary, B and C sit at the apex of the CMRR design process: B defines the operational ambition, C filters the implementable solution space, and A and D are co-optimized within the envelope they jointly establish. The key practical implication is that permissibility assessment should be integrated from the earliest phases of autonomy and communications architecture definition, and specifically to determine whether IS-certified hardware can support the target LoA before physical and sensor design commitments are made. Treating C as a late-stage compliance check risks discovering fundamental hardware ceilings in compute, bandwidth, or energy only after significant investment in A and D has been committed.

#### 5.1.4 Multi-dimensional taxonomy of reviewed CMRRs

**FIGURE 6:**
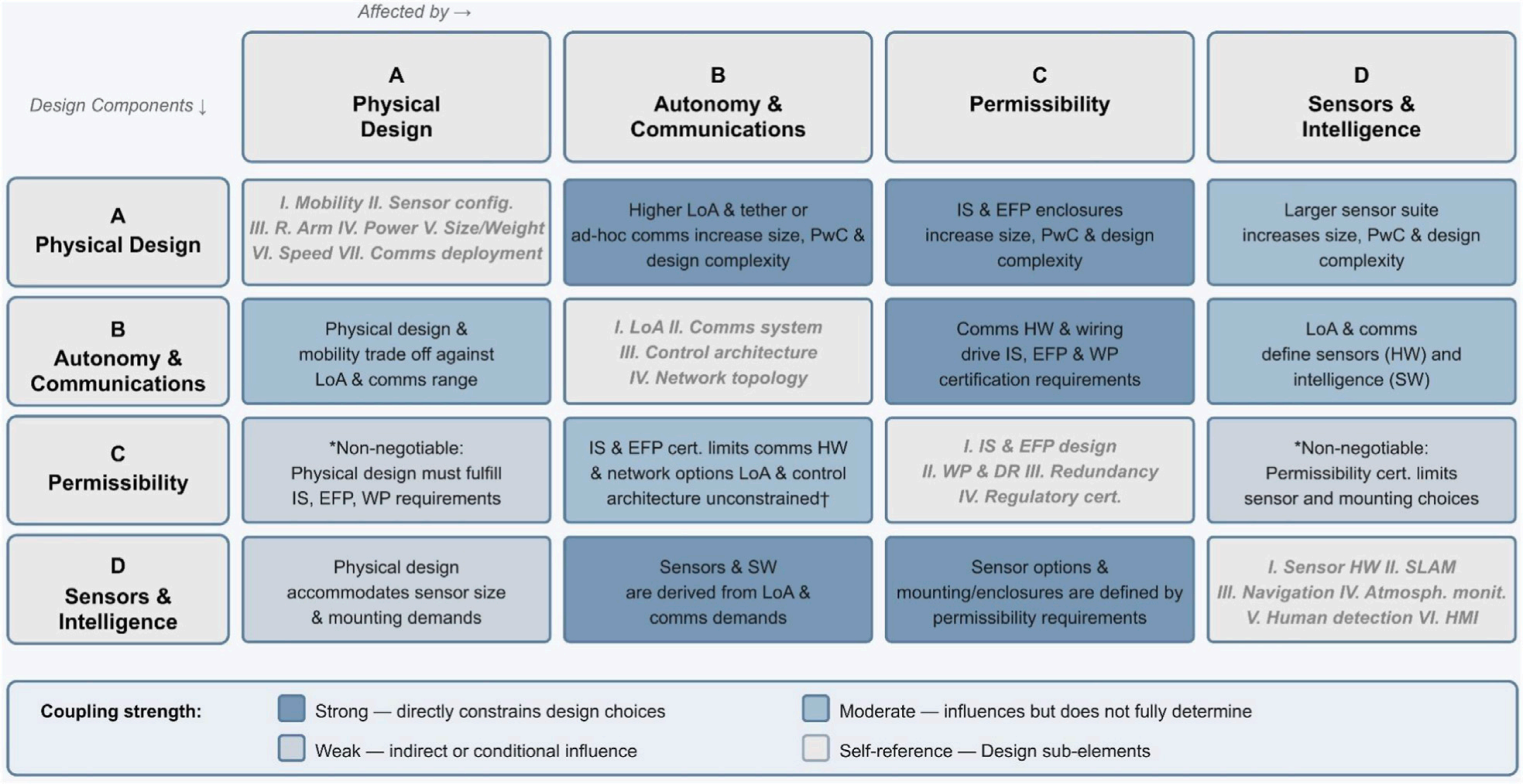
Trade-off matrix of inter-category design constraints for CMRRs. Read across a row to identify which categories affect a given design component; read down a column to identify which design components are affected by a given category. (Abbreviations: R. Arm, robotic arm; LoA, level of autonomy; PwC, power consumption; IS, intrinsically safe; EFP, explosion- and fire-proof; WP, waterproof; HW, hardware; SW, software; DR, dust resistance).

The reviewed robots exhibit a variety of different designs, physical and computational capabilities, and levels of permissibility. Despite various robotic platforms theoretically suitable for mine rescue, only a few have been deployed, influenced by technical, regulatory, and economic factors. The reviewed platforms can be commonly grouped into “early” and “modern” generations based on development decade. The chronological split captures broad technological progress. The development of the early MRRs focused on the speedy deployment of retrofitted platforms originally developed for other domains (e.g., bomb disposal and military reconnaissance), whereas modern MRRs emphasized purpose-built safety and functionality for the harsh conditions of potentially explosive mine atmospheres, uneven terrain, and water ingress. However, a more informative classification frames each CMRR along three independent axes: (A) autonomy level, (B) explosion-protection rating, and (C) primary functional scenario (Figure 6).

**FIGURE 7:**
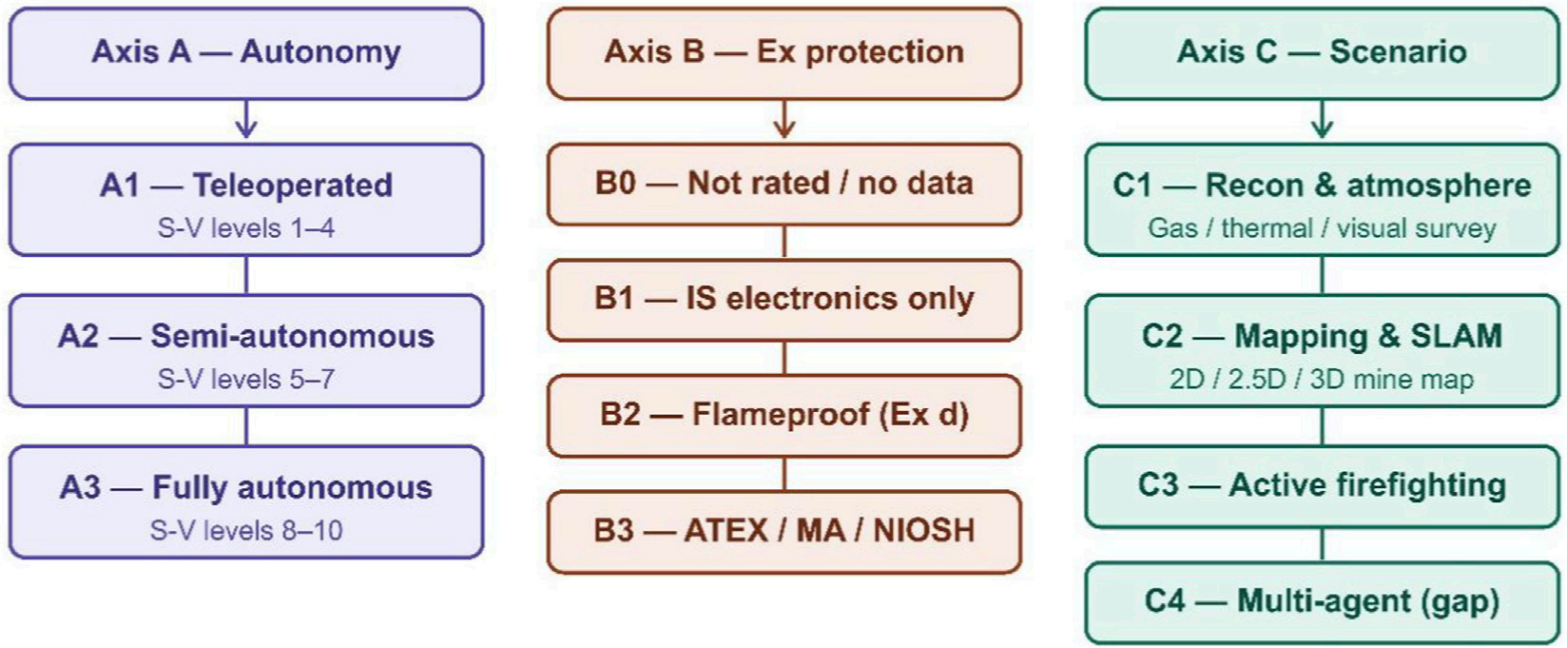
Three-axis classification framework applied to reviewed CMRRs. Autonomy level, mapped to the Sheridan–Verplank (S–V) scale (see Table 1).

Axis A: Autonomy level maps each platform to the Sheridan–Verplank scale already adopted in this review (Table 1). Three bands are used: A1 teleoperated (Levels 1–4, operator retains all decision authority); A2 semi-autonomous (Levels 5–7, shared authority with automated execution of sub-tasks); and A3 fully autonomous (Levels 8–10, onboard decision making without operator approval). Of the 14 reviewed platforms, 12 are classified A1, one achieves A2 (CUMT-V), and one achieves A3 (Groundhog/Cave Crawler).

Axis B: Explosion-protection rating records the highest certified protection standard documented in the primary sources for each platform: B0 not rated or no published data; B1 intrinsically safe (IS) electronics only; B2 flameproof enclosure (Ex d); B3 full system certification (ATEX Group I Category M1, Mining Approval, or NIOSH permissible, as applicable to the platform’s country of origin). Where a platform achieved certification under multiple regional standards (e.g., TeleRescuer: ATEX M1), the highest equivalent band is assigned. Five of the 14 platforms carry a B0 rating, meaning no explosion-protection standard is reported in their primary sources.

Axis C: The primary functional scenario records the principal deployment role reported by platform developers: C1 reconnaissance and atmospheric monitoring (gas sensing, thermal imaging, visual survey); C2 mapping and SLAM (2D/2.5D/3D geometric mapping as the primary deliverable); C3 active firefighting (onboard suppression system); C4 multi-agent coordination (designed for simultaneous multi-robot deployment). Platforms covering more than one scenario receive compound codes (e.g., C1+C2). Ten of the 14 platforms are classified as C1 only, while no platform demonstrates C4.

Table 7 applies this taxonomy to all 14 reviewed CMRRs (KQR48 was excluded due to insufficient published data). Each platform is assigned a three-part code (e.g., ANDROS Wolverine V2: A1-B3-C1).

**TABLE 7:** Three-axis taxonomy of reviewed CMRRs. (Classification applied to 14 reviewed CMRRs; KQR48 excluded due to insufficient published data). Axis A follows the Sheridan–Verplank scale (Table 1).

CMRR (year)	Axis A (autonomy)	Axis B (ex protection)	Axis C (scenarios)	S&R (missions)
Early platforms (≤2005)				
RATLER (1991–95)	**A1**	**B0**	**C1**	No
Numbat (1990–2001)	**A1**	**B3**	**C1**	No
ANDROS Wolverine/V2 (2001–06)	**A1**	**B3**	**C1**	Yes ×7
Groundhog and Cave Crawler (2004–06)	**A3**	**B2**	**C2**	No
Modern platforms (2006 – present)				
Souryu-V (2000–2010)	**A1**	**B0**	**C1**	No
Inuktun VGTV (2000s)	**A1**	**B0**	**C1**	Yes ×2
WA Water Service Robot (c. 2010)	**A1**	**B3**	**C1**	Yes^†^
Subterranean robot (early 2010s)	**A1**	**B0**	**C1**	No
Leader (c. 2016)	**A1**	**B0**	**C1**	Yes
Gemini-Scout (2008–16)	**A1**	**B3**	**C1**	No
MINBOT-II (c. 2014)	**A1/A3** ^‡^	**B2**	**C1**	No
CUMT-V (2006–16)	**A2**	**B2**	**C1+C3**	No
MPI/KRZ-I (2008–16)	**A1**	**B2**	**C1+C2**	No
TeleRescuer (2014–17)	**A1**	**B3**	**C1+C2**	No

A1, levels 1–4 (teleoperated); A2, levels 5–7 (semi-autonomous); A3, levels 8–10 (fully autonomous). Axis B records the highest explosion-protection standard reported in primary sources: B0, not rated or no published data; B1, intrinsically safe electronics; B2, flameproof enclosure (Ex d); B3, full system certification (ATEX/MA/NIOSH permissible). Axis C records the primary functional scenario: C1, reconnaissance and atmospheric monitoring; C2, mapping and SLAM; C3, active firefighting; C4, multi-agent coordination (no current platform). Compound codes (e.g., C1 + C2) indicate multiple documented scenarios.

^†^ WA Water Service Robot was destroyed during its single deployment.

^‡^ MINBOT-II supports switchable A1 and A3 modes; no intermediate A2 behavior is reported by the developers. KQR48 excluded due to insufficient published data.

Three findings emerge directly from this classification. First, 12 of 14 platforms are classified A1 (teleoperated); only Groundhog/Cave Crawler achieves A3, and only CUMT-V reaches A2, confirming that autonomous navigation in coal mine conditions remains an unsolved problem. Second, five platforms carry a B0 rating because the available sources do not report any explosion-protection standard. This constitutes a transparency and reproducibility gap: without certified Ex ratings, independent verification of field readiness is impossible, and procurement bodies cannot make evidence-based comparisons. Future CMRR publications should report the applicable national or international standards, the protection method (IS, Ex d, pressurization, etc.), and the certification body as minimum metadata. Third, 10 of 14 platforms classify as C1 only; a single platform (CUMT-V) achieves C3; and no reviewed platform demonstrates C4 multi-agent coordination.

The design aspects that have received attention in all reviewed CMRRs are i) locomotion that allows flexible and reliable mobility on harsh terrain (A.I), ii) a robust communications system, whether wireless or cable-based (B.II), iii) explosion-, flame-, and waterproofing (C.I and C.II), and iv) sensor suite selection for atmospheric conditions monitoring (D.IV). In more recently developed CMRRs, developers also report on autonomy level (B.I) in conjunction with control system and human–machine interface design (D.VII). Mapping (D.II) or path planning and autonomous navigation (D.III) are usually briefly discussed or omitted, which is consistent with the A1 dominance observed in Table 7. The focus of performance testing is on the clarity and consistent functionality of visual sensors rather than on onboard navigation stacks.

### 5.2 Emerging technologies and research directions

Current research trends in CMRR design can be informed by developments in broader fields such as disaster robotics, planetary exploration robotics, and underground mapping systems. Additionally, rapid advances in electronics, such as miniaturized sensors, energy-efficient processors, and specialized materials, continue to expand the technological possibilities for mine rescue robotics.

The persistent challenges in CMRR deployment identified in Section 5.1, combined with the unique constraints of underground coal mine rescue environments, reveal several research directions that warrant investigation for developing more capable CMRR systems. While general advances in robotics, sensors, and AI contribute to CMRR development, research specifically addressing the constraints of explosive atmospheres, extreme confinement, GPS-denied navigation, and time-critical personnel detection may offer particularly valuable insights. The following discussion examines representative technological trends and research areas that show promise for coal mine applications:

- Mobility issues: Mobility issues stem primarily from weaknesses in mechanical elements such as motors or “walking” mechanisms. Innovations in wheel and track design ranging from flexible metal wheels to deformable hybrid walking mechanisms and bio-inspired locomotion provide suitable solutions (Lopez-Arreguin and Montenegro, 2020; Zou et al., 2020; Zhao et al., 2021). Additionally, the future MRR will integrate the capability of switching between different modes. Robots that can switch between different modes of movement (ground, air, water) to overcome varied terrain challenges will be better equipped for navigating unpredictable environments in rescue operations. Coal mine-specific mobility research should prioritize: (1) propulsion systems optimized for low-ceiling environments with ability to flatten or compress for passage through roof falls, utilizing shape-morphing soft robotics moving with worm-like contractions (Sebastian, 2025), bio-inspired designs like the Scorpio robot that transitions between walking and rolling modes (Martz et al., 2020), and low-profile platforms such as the HADES robot developed to navigate between wall and roof rubble in abandoned mines (Molyneaux et al., 2015; Martz et al., 2020); (2) amphibious locomotion capable of transitioning between flooded sections and dry terrain (while maintaining explosion-proof sealing), as mines frequently transition from dry floors to deep mud or underwater sections. Potential solutions could follow design approaches such as the CSIR-CMERI platform combining tracks with thrusters (Martz et al., 2020), 6 × 6 buoyant crawlers for water-to-rocky terrain transitions (Martz et al., 2020), and explosion-proof motors as demonstrated in the Wolverine robot (Murphy et al., 2009; Martz et al., 2020); (3) climbing mechanisms for traversing ventilation shafts, steep coal seam inclinations (>45° slopes) (Sebastian, 2025), and debris piles typical of post-explosion environments (Martz et al., 2020), employing quadrupedal robots designed for mining conditions (Konieczna-Fulawka et al., 2025), hybrid whegs (spoked wheels with leg-like navigation) (Molyneaux et al., 2015; Martz et al., 2020), and multimodal aerial-ground systems for mapping underground structures including shafts (Konieczna-Fulawka et al., 2025); and (4) adaptive traction systems that can handle the transition from hard rock floors to soft coal dust accumulations or sticky mud without track/wheel fouling, utilizing anisotropic friction feet for stability (Sebastian, 2025), whegs that avoid entrapment where traditional wheels fail, and buoyant wheels that prevent immobilization in mud (Martz et al., 2020). Furthermore, research into fail-safe mobility modes that allow partial functionality after component damage (e.g., continued operation with damaged tracks or wheels) is essential for extending mission duration in high-risk environments. For example, the Omnitread robot’s omnidirectional tracks maintain locomotion after rollover (Martz et al., 2020), self-healing hydrogel materials enable autonomous damage repair (Sebastian, 2025), and distributed consensus algorithms ensure multi-robot mission continuity despite node failures (see item d below).
- Communications: Wireless communications are a highly sought-after goal for underground environments. Although creating robust and reliable wireless networks in narrow tunnel-like environments remains extremely challenging, abundant research initiatives explore new, efficient wireless sensors for tunnel-like environments or ways of deploying and maintaining *ad hoc* wireless networks in harsh environments such as smart sensor node networks or swarms of robotic agents (Ranjan et al., 2020; Diaz et al., 2019; Sadeghi et al., 2022; Petrlik et al., 2022). Techniques like mobile *ad hoc* networks (MANETs) and breadcrumb communication are widely investigated and could be useful in maintaining connectivity in mine rescue missions (Queralta et al., 2020). New forms of long-range wireless communications, such as through-the-earth (TTE) communication, which uses ultra-low frequency electromagnetic signals to penetrate solid rock and earth, are mentioned as a promising technology for ensuring communication between robots and rescue teams in underground environments, as well. This is important for maintaining control over the robot and ensuring data are transmitted back to the operators (Martz et al., 2020). Specific research priorities for coal mine rescue communications include (1) through-the-earth (TTE) systems utilizing ultra-low frequency signals (3,000–8,000 Hz) optimized for the electromagnetic properties of coal seams and typical overburden depths (100–500 m), with promising approaches including magnetic induction (MI) communication and magnetoelectric (ME) antenna technologies that can achieve significant wavelength reduction, potentially enabling ultra-miniaturized components capable of bidirectional communication at depths from 310 m to more than 470 m on battery-powered robots (Luo et al., 2024; Ma et al., 2025); (2) characterization and mitigation of signal degradation in post-explosion environments where smoke, airborne coal dust, high humidity, and ionized gases severely impair conventional radiofrequency (RF) propagation, with research needed to address challenges such as MI fast fading due to antenna vibrations and orientation changes as mobile robots navigate rough terrain (Ma et al., 2025; He et al., 2024); (3) autonomous communication node placement algorithms that could utilize approaches such as random-search optimization and distributed consensus protocols to account for complex drift geometry, pillar spacing patterns, and roof fall probabilities, with the goal of establishing reconfigurable mesh networks that remain robust despite individual node failures and can dynamically bypass obstructed pathways to maintain connectivity during extended missions (Konieczna-Fulawka et al., 2025; Ma et al., 2025); and (4) development of hybrid communication architectures, with recent research exploring integration of coal mine networks into space-air-ground-underground (SAGUI) frameworks that enable seamless transitions between high-throughput Wi-Fi mesh networks (IEEE 802.11s) for local high-bandwidth transmission and long-range, energy-efficient protocols like LoRaWAN for extended coverage, using real-time assessment of link quality to determine optimal mode selection and trigger emergency tether deployment when wireless options fail (Saxena and Shukla, 2023; Huang et al., 2024). Research into communication protocols optimized for high-latency, intermittent-connectivity scenarios typical of deep underground environments remains critically important, with delay-tolerant networking (DTN) approaches showing promise through store-carry-and-forward (SCF) mechanisms where mobile robots carry data across connectivity gaps, supported by onboard data buffers that store critical information during outages, while lightweight protocols such as MQTT, CoAP, and WebRTC offer potential solutions for real-time telemetry and video streaming despite severe bandwidth and latency constraints (Konieczna-Fulawka et al., 2025; Koukis et al., 2024).
- Sensors and intrinsic safety: Rapid advancements in sensor technology and computational power have transformed robots from simple decision-making devices into fully autonomous systems equipped with artificial intelligence and emerging digital twin capabilities for real-time scenario simulation and predictive intelligence. The miniaturization of OEMs through micro-electromechanical systems (MEMS) fabrication and the increasing number of manufacturers dedicated to self-driving vehicle sensor development provide efficient and compact rugged sensors that are designed to function in harsh conditions, with materials such as silicon carbide (SiC) showing promise for high-temperature and high-pressure applications (Liu Q. et al., 2025; Cacciuttolo et al., 2023). Microfabrication techniques have proven essential for creating miniaturized, cost-effective sensors that minimize power consumption while remaining accessible for widespread deployment (Saxena and Shukla, 2023). Inherently intrinsically safe sensor technologies, such as optical fiber systems that are immune to electromagnetic interference and corrosion, have the potential to reduce design needs in terms of housing and positioning of sensors, although formal certification for explosive environments remains an ongoing challenge for many state-of-the-art technologies (Duarte et al., 2022; Abbasi et al., 2021). At the same time, advances in sensor abilities, resolution, and accuracy will assist MRR designers in tackling intelligence requirements. For coal mine rescue applications specifically, critical sensor research priorities include (1) development of intrinsically safe multi-gas sensors capable of simultaneously detecting methane, carbon monoxide, hydrogen sulfide, and oxygen deficiency at parts-per-million sensitivity while maintaining explosion-proof certification, with emerging approaches utilizing 2D nanostructures (e.g., MXenes or MoS_2_), graphene-based sensors demonstrating detection capabilities below 10 ppb for nitrogen dioxide, or CMOS-compatible floating-gate field-effect transistors potentially achieving parts-per-billion (ppb) sensitivity for early gas detection, as demonstrated by nanocrystalline polyaniline-based nanocomposites that selectively detect carbon monoxide at ppb levels (Saxena and Shukla, 2023; Hooshmand et al., 2023); (2) robust particulate matter sensors that can differentiate coal dust from other airborne contaminants in post-explosion environments, with techniques such as computer-controlled scanning electron microscopy with energy dispersive X-ray analysis (SEM-EDX) or aerodynamic spectrometers offering potential solutions for analyzing dust morphology and chemical composition (Abbasi et al., 2021); (3) thermal and infrared sensors with enhanced penetration capabilities for detecting trapped personnel through smoke, dust, and debris, potentially integrated into thermal-inertial odometry frameworks to enable simultaneous victim detection and robot navigation in visibility-impaired environments (Liu H. et al., 2025); and (4) miniaturized environmental sensor arrays that can create high-resolution 3D maps of atmospheric conditions (temperature, humidity, airflow patterns) to assess re-entry safety for human rescue teams, with research exploring multimodal sensor fusion approaches combining LiDAR, inertial measurement units (IMUs), and ultra-wideband (UWB) positioning to ensure robust localization in GPS-denied tunnel environments (Duarte et al., 2022; Liu H. et al., 2025). The development of self-cleaning sensor mechanisms resistant to coal dust fouling remains a fundamental challenge requiring focused research attention, with biomimetic approaches such as superhydrophobic surfaces inspired by the lotus effect showing promise for creating anti-fouling and anti-corrosive barriers that prevent sensor degradation in harsh underground conditions (Dai et al., 2024).
- Automation and intelligence: The evident hype of the latest years regarding self-driving vehicles and robotics has led to multi-faceted advances in sensors, processors, software, and robotic deployment frameworks that will critically shape the future of MRRs. Crucial technologies that are or can be employed in disaster and rescue robotics include
- Swarm robotics deployment algorithms and collaborative multi-agent robotic systems. Multi-robot systems in S&R operations can significantly improve rescue efforts by providing faster response times, situational awareness, and real-time mapping. The DARPA Subterranean Challenge showed this approach that very few of the reviewed CMRRs have followed, that is, the collaboration of multiple robotic agents toward fulfilling the mission of exploring the environment. The superiority of this approach lies in the extreme flexibility that a multi-agent robotic system allows for designing and executing a mission. This approach allows for building a great variety of robotic agents, each type equipped with different locomotion systems, sensor suites, and intelligence. Aerial, ground, and underwater robots collaborate to improve the efficiency of S&R missions. The reduced complexity in designing and building such a decentralized robotic system allows for fulfilling all the design requirements. Moreover, the deployment of such a system is characterized by significantly increased time and functional performance, and eliminating the disadvantage of a single-point failure. (Queralta et al., 2020; Szczurek et al., 2023; Chen et al., 2022; Huang et al., 2024).
- SLAM, autonomous navigation, and path planning in GPS-denied underground environments. SLAM and autonomous navigation represent the core enabler for transitioning CMRRs from teleoperation toward autonomous operation. The DARPA Subterranean Challenge (DARPA, 2022) remains the most comprehensive open evaluation of autonomous mapping and navigation in GPS-denied underground conditions to date, and the literature it has generated provides the recommended entry point for developers targeting higher autonomy levels in future CMRR platforms. For large-scale subterranean mapping, LiDAR-centric SLAM with robust back-ends and distributed inter-robot loop closures represents the most mature and field-validated approach, as demonstrated by systems such as LAMP, DOOR-SLAM, Kimera-Multi, and the SKiD-SLAM family (Ebadi et al., 2020; Chang et al., 2022; Kim et al., 2022). A practical underground exploration system typically employs a LiDAR-based front-end with IMU fusion, complemented by UWB beacons or wheel odometry to constrain drift in long featureless corridors (Liu Q. et al., 2025; Ebadi et al., 2020; Kim et al., 2022; Hao et al., 2025; Inostroza et al., 2023). Vision-based SLAM remains relevant as a complement in resource-constrained agents or where lighting permits but tends to be less robust than LiDAR under the severe perceptual degradation of mine environments, and a hybrid multi-sensor approach is commonly adopted (Kim et al., 2022; Debeunne et al., 2023). Coal mine conditions impose constraints beyond those of general subterranean environments: the repetitive and featureless geometry of mine drifts challenges loop closure detection, airborne coal dust degrades LiDAR returns and camera images, and dynamic roof collapse invalidates previously built maps. Degeneracy-aware front-ends such as DARE-SLAM address perceptual aliasing in feature-sparse zones and help avoid erroneous loop closures and map distortion (Ebadi et al., 2021). For heterogeneous robot fleets operating under bandwidth limitations, distributed cooperative SLAM with selective data sharing and pairwise consistency maximization-based loop closure consistency offers a scalable solution while maintaining mapping fidelity (Chang et al., 2022; Kim et al., 2022; Agha et al., 2022). Path planning in this context requires replanning algorithms capable of operating within the computational envelope of IS-certified embedded processors, a constraint that standard approaches developed for unconstrained hardware do not address. Matching the algorithmic demand of SLAM and path planning to what IS-certified onboard compute can support remains the most coal mine-specific research gap in this domain.
- Machine learning /artificial intelligence (ML/AI) in image processing for detection of humans, spaces, or objects. The integration of hybrid sensor configurations (RGB, thermal, and LiDAR data fusion) has emerged as essential to overcome the severe perception degradation caused by low illumination, smoke, and airborne dust in underground environments (Liu H. et al., 2025; Imam et al., 2023). Such algorithms help identify “hot spots” or regions of interest (ROIs) where humans may be present, even in challenging situations like people being obscured or lying down, while also potentially identifying geotechnical hazards such as roof fall risks, with recent research demonstrating detection accuracy and recall rates approaching 90% (Lopez et al., 2024). The integration of LiDAR, thermal imaging, and augmented reality (AR) for real-time 3D mapping could improve perception in pitch-black or smoke-filled environments (Kot et al., 2016; Demirkan et al., 2024; Szrek et al., 2020; Cruz Ulloa et al., 2022). For example, frameworks use thermal radiation information to enable precise robot navigation in textureless environments where visible light cameras fail (Liu H. et al., 2025). AI and data processing research specific to coal mine rescue should address: (1) real-time atmospheric hazard assessment algorithms that fuse multi-sensor data (gas concentrations, temperature, airflow) to predict explosion risks, oxygen-deficient zones, and safe entry corridors for rescue teams; (2) machine learning models for victim detection, with approaches such as YOLO (you-only-look-once) optimized for high-speed detection and trained on the unique signatures of humans in coal mine environments (thermal signatures may be obscured by ambient heat, visual detection is challenged coal dust, and acoustic detection must discriminate machinery noise) (He et al., 2024; Imam et al., 2023); (3) autonomous decision-making frameworks that prioritize exploration paths based on probability of survivor location, atmospheric safety, and structural stability, with research exploring graph neural networks to evaluate loop closure candidates that can significantly reduce pose uncertainty during autonomous navigation (Wang et al., 2025); and (4) data compression and prioritization algorithms optimized for low-bandwidth communication links, utilizing lightweight feature descriptors (e.g., Scan Context or LiDAR-Iris) to transmit representative summaries rather than raw point clouds, ensuring critical information (victim location, imminent hazards) reaches command centers despite intermittent connectivity (Wang et al., 2025). The development of explainable AI (XAI) systems that can justify autonomous decisions to rescue commanders represents a crucial research gap for enabling human–robot teaming in high-stakes rescue scenarios (Bashu and Srikanth, 2025).
- Wearable devices and IoTs to provide georeferenced or human-specific real-time data. The integration of wearable devices specifically tailored for miners, such as smart helmets and smartwatches, can provide valuable data on trapped personnel health, including heart rate, blood oxygen saturation (SpO_2_), body temperature, and EEG signals (Sadeghi et al., 2022; Adjiski et al., 2019; Mardonova and Choi, 2018; Lee and Chien, 2020; Singh et al., 2022; Svertoka et al., 2021; Liang et al., 2024). These devices are evolving into multimodal platforms combining physiological monitoring with environmental hazard detection and integrating gas sensors for carbon monoxide, nitrogen dioxide, and hydrogen sulfide using 2D nanostructures (e.g., MXenes or MoS_2_) capable of ppb-level sensitivity. To manage data volumes from continuous monitoring, research is advancing edge computing and on-node processing for real-time signal analysis and sensor fusion directly on wearable devices (Hooshmand et al., 2023). To ensure functionality in harsh underground conditions with high humidity, coal dust, and corrosive substances, research is exploring biomimetic superhydrophobic surfaces inspired by the lotus effect to create self-cleaning, anti-fouling, and anti-corrosive barriers that prevent sensor degradation (Dai et al., 2024).
- Advanced human–machine interfaces are needed to enhance teleoperation performance by providing better interaction and feedback through increased data flow, improving decision making and control. Sophisticated human–machine interface design that encompasses virtual reality tools will be implemented for enhancing situational awareness, data visualization, and real-time decision making (Szczurek et al., 2023; Chen et al., 2022; Opiyo et al., 2021).

## 6 Conclusion

This review has examined CMRRs developed and deployed for disaster response in underground mines over the last 3 decades, accumulating applications from international literature to establish a collective overview of desirable requirements for (coal) mine rescue robots. The reviewed sources consistently highlight the absence of commonly accepted development protocols and specifications, or in-depth measurable data regarding performance evaluation of the different design elements. The mining-specific literature skews heavily toward hardware selection with a focus on specific design aspects and provides only partial performance analysis.

Three decades of CMRR development have produced a consistent pattern. The earliest operationally deployed platforms, such as Leader (Wang et al., 2018; RT News, 2016), the WA Water Service Company robot (Wang et al., 2018; Reuters, 2010), and ANDROS Wolverine (Murphy et al., 2009), were retrofitted from general-purpose platforms (bomb disposal, pipeline inspection, military reconnaissance), with modifications focused on explosion-proof retrofitting and gas detection sensor suites. Most failed in the field on fundamental specifications: inadequate waterproofing, short tether range, limited terrain mobility, or tether breakage. (Wang et al., 2018; RT News, 2016) (Wang et al., 2018; Reuters, 2010). ANDROS Wolverine is the only platform with a documented deployment record comprising four successful or partially successful operations of seven total deployments between 2001 and 2006 (Murphy et al., 2009). Moreover, a subset of the early MRRs feature designs that focused on improving a specialized characteristic or functionality, rather than designing a fully functional MRR. These robots are notably smaller and lighter and focus on exploration and mapping, that is, Groundhog, Cave Crawler, ATRV, Souryu series, and Subterranean Robot. Modern MRRs developed from the late 2010s onward (Gemini-Scout, MINBOT-II, the CUMT series, MPI, and TeleRescuer) have reversed these priorities: they are purpose-built, carry certified explosion-proof and waterproof designs, and integrate multimodal sensor suites with partial autonomous capability. However, none has been operationally deployed or exhaustively evaluated in mine emergency scenarios.

The reviewed platforms are almost exclusively single robotic agents, and the discussion has focused accordingly on their mechanical and computational components. This scope matters for interpretation because individual design elements are not independent. The interdependencies among design-requirement categories, and in particular, the co-defining relationship between autonomy architecture and permissibility, are discussed extensively in Section 5.1.3. In brief, the permissibility requirements (C-type) must be satisfied by both physical design (A-type) and communications and autonomy (B-type) requirements; sensor and intelligence requirements (D-type) are interrelated with B-type; navigation/path planning (D.III) must account for physical design characteristics (A-type); and HMI design (D.VII) requires inputs from A- and B-type requirements as well as D.II–D.VI. Consequently, while components such as A.IV, C.I, and C.II can be evaluated in isolation, overall robot performance can only be meaningfully assessed when the complete platform is tested under realistic deployment scenarios. The absence of standardized performance requirements is the most consequential structural gap in the field. The available standards address component-level permissibility (electrical equipment design, installation, and maintenance), but there are no guidelines on functional performance (minimum mapping range, sensor accuracy under dust and smoke, autonomous recovery from communication loss, or the format of data relayed to a surface command center). Without such standards, each development team defines its own evaluation criteria, making cross-platform comparison impossible and precluding the accumulation of the evidence base needed to build industry-wide design consensus. The evaluation framework used in the DARPA Robotics Challenge (Pratt and Manzo, 2013) and the DARPA Subterranean Challenge (RoboCup Federation, 2023), which are based on task- and subtask scoring under realistic deployment scenarios, provides a starting model that could be adapted to coal mine-specific scenarios.

The most reported challenges or failures of CMRR performance can be categorized into four groups:

- communication and tether failures (B.I, B.II): limited range, tether breakage, and entanglement problems (appear across the widest number of reviewed platforms);
- mobile mechanism failures (A.I, A.V): track separation, inability to traverse specific obstacle configurations, and challenges in maneuvers due to size and weight (second most frequently reported);
- perception failures (A.III, D.I): noise and interference in sensor data, camera glare, limited visibility due to mud, dust, or poor illumination, and challenges in teleoperation due to limited awareness (appear in several platforms);
- electrical or power failures (A.IV, C.I–C.III): voltage loss, battery depletion, and component failures under harsh environmental conditions (smaller subset).

Notably, there are no reports regarding the requirements of identifying fires (D.V) or recognizing humans (D.VI) from the reviewed sources. Both these requirements have been investigated in recent years using thermal and visual cameras with machine learning to recognize these features of interest.

Building on the lessons identified in this review, the following research priorities are recommended for future CMRR development:

1. Intrinsically safe sensor arrays (D.I–D.III): develop multi-gas sensors with coal dust resistance and ppb-level sensitivity, certified for explosive atmospheres, integrated with self-cleaning mechanisms for sustained underground operation.
2. Adaptive locomotion systems (A.I–A.III): advance low-profile, shape-morphing, and amphibious locomotion capable of navigating low-ceiling, debris-laden, and flooded mine sections, including fail-safe mobility modes for partial operation after component damage.
3. Through-the-earth and hybrid communication (B.I–B.II): develop TTE/MI wireless modules optimized for coal seam electromagnetic properties and overburden depths, integrated within hybrid architectures that fall back to a wired tether when wireless links fail.
4. AI-based hazard assessment and navigation (B.III–B.IV, D.IV–D.VII): advance real-time atmospheric hazard prediction, dust-robust sensor fusion for SLAM in GPS-denied environments, and explainable autonomous decision making for human–robot teaming in rescue operations.
5. Standardized testing and failure reporting (A–D): establish a CMRR-specific test course and structured field-reporting protocol, aligned with DARPA SubT metrics, to enable systematic failure-mode data collection across future deployments and inform evidence-based reliability design.

Successful mine rescue operations ultimately require moving beyond stand-alone platforms toward collaborative multi-agent systems capable of distributed sensing, communication, and decision making, as demonstrated by the DARPA Subterranean Challenge (RoboCup Federation, 2023).

In conclusion, despite the declining frequency of severe mine emergencies, driven by improvements in safety measures, remote operations, and personnel self-rescue options, the need for capable CMRRs remains an essential element of proactive mine preparedness and a critical means of protecting human life.

## References

[B1] Abbasi B. Wang X. L. Chow J. C. Watson J. G. Peik B. Nasiri V. (2021). Review of Respirable Coal Mine Dust Characterization for Mass Concentration, Size Distribution and Chemical Composition. Minerals 11 (4), 426. 10.3390/min11040426

[B2] Ackerman E. (2023). Your Guide to the Darpa Subt Finals: Meet the Teams: How the Eight Systems Track Teams Are Approaching the Final Event. Available online at: https://spectrum.ieee.org/darpa-subterranean-challenge (Accessed September 20, 2023).

[B3] Adjiski V. Despodov Z. Mirakovski D. Serafimovski D. (2019). System Architecture to Bring Smart Personal Protective Equipment Wearables and Sensors to Transform Safety at Work in the Underground Mining Industry. Rudarsko-Geolosko-Naftni Zb. 34 (1), 37–44. 10.17794/rgn.2019.1.4

[B4] Agha A. Otsu K. Morrell B. Fan D. D. Thakker R. Santamaria-Navarro A. (2021). Nebula: Quest for Robotic Autonomy in Challenging Environments; Team Costar at the Darpa Subterranean Challenge.arXiv Preprint arXiv:2103.11470. 10.48550/arXiv.2103.11470

[B5] Agha A. Otsu K. Morrell B. Fan D. D. Thakker R. Santamaria-Navarro A. (2022). Nebula: Team Costar's Robotic Autonomy Solution That Won Phase Ii of Darpa Subterranean Challenge. Field Robot. 2, 1432–1506. 10.55417/fr.2022047

[B7] Arai M. Tanaka Y. Hirose S. Kuwahara H. Tsukui S. (2008). Development of “Souryu-Iv” and “Souryu-V”: Serially Connected Crawler Vehicles for in-Rubble Searching Operations. J. Field Robotics 25 (1-2), 31–65. 10.1002/rob.20229

[B8] Attoh-Okine N. Bullock D. M. (2000). Automation in Transportation System Construction and Maintenance Environmental Life Cycle Assessment View Project Machine Learning View Project.

[B9] Baker C. Morris A. Ferguson D. Thayer S. Whittaker C. Omohundro Z. (2004). “A Campaign in Autonomous Mine Mapping,” in *Proceedings - IEEE International Conference on Robotics and Automation*. 2004 (Piscataway, NJ, United States: IEEE). 10.1109/robot.2004.1308118

[B10] Bashu B. Srikanth B. (2025). Artificial Intelligence Enabled Wireless Sensor Network for Underground Mines Safety: A Systematic Review. J. Institution Eng. (India) Ser. D, 1–17. 10.1007/s40033-025-00971-1

[B11] Bołoz Ł. Biały W. (2020). “Automation and Robotization of Underground Mining in Poland,” in Applied Sciences (Switzerland) (Basel, Switzerland: MDPI AG), 1–14. 10.3390/app10207221

[B12] Brnich M. Kowalski-Trakofler K. M. Brune J. (2010). Underground Coal Mine Disasters 1900-2010: Events, Responses, and a Look to the Future. Extr. Science A Century Mining Research, 363–372. Available online at: https://stacks.cdc.gov/view/cdc/227216 (Accessed August 16, 2023).

[B13] Cacciuttolo C. Guzman V. Catrinir P. Atencio E. Komarizadehasl S. Lozano-Galant J. A. (2023). Low-Cost Sensors Technologies for Monitoring Sustainability and Safety Issues in Mining Activities: Advances, Gaps, and Future Directions in the Digitalization for Smart Mining. Sensors (Basel) 23 (15), 6846. 10.3390/s23156846 37571628 PMC10422650

[B14] Calder W. Snyder D. P. Burr J. F. (2018). “Intrinsically Safe Systems: Equivalency of International Standards Compared to U.S. Mining Approval Criteria,” in *IEEE Transactions on Industry Applications*. 2018 (Piscataway, NJ, United States: IEEE). 10.1109/TIA.2018.2804322 PMC6275100 30514985

[B15] Callow D. S. (2018). *Mine Rescue Robotics: Gemini Scout*. 2018. Albuquerque, NM (United States): Sandia National Lab.SNL-NM.

[B16] Casper J. Micire M. Li Gang R. (2004). “Inuktun Services Ltd.–Search and Rescue Robotics,” in Proceedings of the ICCE 3rd International Conference on Continental Earthquakes.

[B17] Caterpillar (2023). Take Command of Your Operation Cat® Minestar™ Command for Hauling. Available online at: https://www.cat.com/en_US/by-industry/mining/surface-mining/surface-technology/command/command-hauling.html (Accessed August 10, 2023).

[B18] Chadeev V. M. Aristova N. I. (2017). Control of Industrial Automation. IEEE.

[B19] Chang Y. Ebadi K. Denniston C. E. Ginting M. F. Rosinol A. Reinke A. (2022). Lamp 2.0: A Robust Multi-Robot Slam System for Operation in Challenging Large-Scale Underground Environments. Ieee Robotics Automation Lett. 7 (4), 9175–9182. 10.1109/Lra.2022.3191204

[B20] Chen S. O'Brien M. J. Talbot F. Williams J. Tidd B. Pitt A. (2022). “Multi-Modal User Interface for Multi-Robot Control in Underground Environments,” in 2022 IEEE/RSJ International Conference on Intelligent Robots and Systems (IROS) (IEEE).

[B21] Cosbey A. Mann H. Maennling N. Toledano P. Geipel J. Brauch M. D. (2016). Mining a Mirage? Reassessing the Shared-Value Paradigm in Light of the Technological Advances in the Mining Sector.

[B22] Cruz Ulloa C. Garcia M. del Cerro J. Barrientos A. (2022). “Deep Learning for Victims Detection from Virtual and Real Search and Rescue Environments,” in Iberian Robotics conference (Springer).

[B23] Cyran K. A. Moczulski W. Myszor D. Paszkuta M. Ruranski A. Kalisch M. (2018). “Immersive Human-Machine Interface for Controlling the Operation of the Telerescuer Robot,” in Proc. of the Fifth Intl. Conf. Advances in Computing, Communication and Information Technology- CCIT 2017. 10.15224/978-1-63248-131-3-28

[B24] Dai Z. Lei M. Ding S. Zhou Q. Ji B. Wang M. (2024). “Durable Superhydrophobic Surface in Wearable Sensors: From Nature to Application,” in Exploration (Wiley Online Library). 10.1002/EXP.20230046 PMC11022629 38855620

[B25] DARPA (2022). Darpa Subterranean (Subt) Challenge (Archived). Available online at: https://www.darpa.mil/program/darpa-subterranean-challenge (Accessed August 9, 2023).

[B26] Davis S. G. Engel D. van Wingerden K. (2015). Complex Explosion Development in Mines: Case Study—2010 Upper Big Branch Mine Explosion. Process Saf. Prog. 34 (3), 286–303. 10.1002/prs.11710

[B27] Debeunne C. Vallvé J. Torres A. Vivet D. (2023). Fast Bi-Monocular Visual Odometry Using Factor Graph Sparsification. IEEE/RSJ International Conference on Intelligent Robots and Systems IROS. 10.1109/iros55552.2023.10341644

[B28] Delmerico J. Mintchev S. Giusti A. Gromov B. Melo K. Horvat T. (2019). The Current State and Future Outlook of Rescue Robotics. J. Field Robotics 36 (7), 1171–1191. 10.1002/rob.21887

[B29] Demirkan D. C. Segal A. Mallik A. Duzgun S. Petruska A. J. (2024). *Real-Time Perception Enhancement in Obscured Environments for Underground Mine Search and Rescue Teams.* AI. Comput. Sci. Robotics Technol. (23). 10.5772/acrt.33

[B30] Deuel J. Salton J. R. Hobart C. G. Garretson J. Callow D. S. (2018). *Mine Rescue Robotics: Gemini Scout-18507*. 2018. Albuquerque, NM (United States): Sandia National Lab.SNL-NM.

[B31] Diaz S. Mendez D. Kraemer R. (2019). A Review on Self-Healing and Self-Organizing Techniques for Wireless Sensor Networks. J. Circuits Syst. Comput. 28 (5), 1930005. 10.1142/S0218126619300058

[B32] Dinelli C. Racette J. Escarcega M. Lotero S. Gordon J. Montoya J. (2023). Configurations and Applications of Multi-Agent Hybrid Drone/Unmanned Ground Vehicle for Underground Environments: A Review. Drones 7, 136. 10.3390/drones7020136

[B33] DoL U. S. MSHA (2021). Unified Mine Rescue Training (Advanced) -Underground Coal and Metal/Nonmetal Mines. Arlington, VA: U.S. Department of Labor, Mine Safety and Health Administration. National Mine Health and Safety Academy.

[B34] Duarte J. Rodrigues F. Castelo Branco J. (2022). Sensing Technology Applications in the Mining Industry-a Systematic Review. Int. J. Environ. Res. Public Health 19 (4), 2334. 10.3390/ijerph19042334 35206524 PMC8872082

[B35] EAC certification for the Eurasian Union (2011). Technical Regulations Cu Tr 012/2011 on the Safety of Equipment in Explosion Hazardous Environments—Certification and Declaration of Conformity.

[B36] Ebadi K. Chang Y. Palieri M. Stephens A. Hatteland A. Heiden E. (2020). Lamp: Large-Scale Autonomous Mapping and Positioning for Exploration of Perceptually-Degraded Subterranean Environments. IEEE International Conference on Robotics and Automation ICRA, 80–86. 10.1109/icra40945.2020.9197082

[B37] Ebadi K. Palieri M. Wood S. Padgett C. Agha-mohammadi A. A. (2021). Dare-Slam: Degeneracy-Aware and Resilient Loop Closing in Perceptually-Degraded Environments. J. Intelligent and Robotic Syst. 102 (1), 2. 10.1007/s10846-021-01362-w

[B38] EUR-Lex (2014). Directive 2014/34/Eu of the European Parliament and of the Council of 26 February 2014 on the Harmonisation of the Laws of the Member States Relating to Equipment and Protective Systems Intended for Use in Potentially Explosive Atmospheres.

[B39] euRobotics (2022). European Robotics League. Available online at: https://eu-robotics.net/eurobotics/activities/european-robotics-league/ (Accessed August 10, 2023).

[B40] EUROSTAT Statistical Books (2010). Health and Safety at Work in Europe (1999-2007): A Statistical Portrait. Belgium: Publications Office of the European Union.

[B41] Faghihi V. Nejat A. Reinschmidt K. F. Kang J. H. (2015). Automation in Construction Scheduling: A Review of the Literature. Int. J. Adv. Manuf. Technol. 81 (9-12), 1845–1856. 10.1007/s00170-015-7339-0

[B42] Farook Z. Matthews C. Skinner M. Brown M. (2019). “Automation in Geotechnical Design - Application and Case Studies,” in Proceedings of the XVII ECSMGE-2019 Geotechnical Engineering Foundation of the Future (London, United Kingdom: International Society for Soil Mechanics and Geotechnical Engineering). 10.32075/17ECSMGE-2019-0971

[B43] Gossett S. (2020). Examples of Rescue Robots You Should Know. Available online at: https://builtin.com/robotics/rescue-robots (Accessed August 10, 2023).

[B44] Green J. (2013). “Mine Rescue Robots Requirements,” in 6th Robotics and Mechatronics Conference (RobMech) (Durban, South Africa).

[B45] Gupta A. K. Arora S. K. (2009). Industrial Automation and Robotics. New Delhi, India: University Science Press.

[B46] Hainsworth D. W. (2001). Teleoperation User Interfaces for Mining Robotics. Aut. Robots 11 (1), 19–28. 10.1023/A:1011299910904

[B47] Hamberg R. Verriet J. (2012). Automation in Warehouse Development. Automation in Warehouse Development. Springer-Verlag London Ltd, 1–241. 10.1007/978-0-85729-968-0

[B48] Hancock P. A. Nourbakhsh I. Stewart J. (2019). On the Future of Transportation in an Era of Automated and Autonomous Vehicles. Proc. Natl. Acad. Sci. U. S. A. 116 (16), 7684–7691. 10.1073/pnas.1805770115 30642956 PMC6475395

[B49] Hao M. Ren J. Ji X. Bi Y. Zhao S. Wu M. (2025). Research on Multimodal Data Enhanced Slam Algorithm for Global Mapping of Underground Coal Mines. Sci. Rep. 15 (1), 37124. 10.1038/s41598-025-21053-y 41131121 PMC12549982

[B50] He B. Ji X. X. Li G. Cheng B. (2024). Key Technologies and Applications of Uavs in Underground Space: A Review. Ieee Trans. Cognitive Commun. Netw. 10 (3), 1026–1049. 10.1109/Tccn.2024.3358545

[B51] Hooshmand S. Kassanos P. Keshavarz M. Duru P. Kayalan C. I. Kale I. (2023). Wearable Nano-Based Gas Sensors for Environmental Monitoring and Encountered Challenges in Optimization. Sensors (Basel) 23 (20), 8648. 10.3390/s23208648 37896744 PMC10611361

[B52] Huang Z. H. Ge S. R. He Y. H. Wang D. D. Zhang S. X. (2024). Research on the Intelligent System Architecture and Control Strategy of Mining Robot Crowds. Energies 17 (8), 1834. 10.3390/en17081834

[B53] Hudson N. Talbot F. Cox M. Williams J. Hines T. Pitt A. (2021). Heterogeneous Ground and Air Platforms, Homogeneous Sensing: Team Csiro Data61's Approach to the Darpa Subterranean Challenge. arXiv Preprint arXiv:2104.09053. 10.48550/arXiv.2104.09053

[B54] IEC (2017). Iec 60079-0:2017: Explosive Atmospheres - Part 0: Equipment - General Requirements. Available online at: https://webstore.iec.ch/publication/32878 (Accessed August 12, 2023).

[B55] Imam M. Baina K. Tabii Y. Ressami E. M. Adlaoui Y. Benzakour I. (2023). The Future of Mine Safety: A Comprehensive Review of Anti-Collision Systems Based on Computer Vision in Underground Mines. Sensors (Basel) 23 (9), 4294. 10.3390/s23094294 37177497 PMC10181612

[B56] Inostroza F. Parra-Tsunekawa I. Ruiz-Del-Solar J. (2023). Robust Localization for Underground Mining Vehicles: An Application in a Room and Pillar Mine. Sensors (Basel) 23 (19), 8059. 10.3390/s23198059 37836889 PMC10574974

[B57] Jung T. Lim J. Bae H. Lee K. K. Joe H. M. Oh J. H. (2018). Development of the Humanoid Disaster Response Platform Drc-Hubo. Ieee Trans. Robotics 34 (1), 1–17. 10.1109/Tro.2017.2776287

[B58] Kasprzyczak L. Szwejkowski P. Cader M. (2016). *Robotics in Mining Exemplified by Mobile Inspection Platform.* Mining–Informatics. Automation Electr. Eng. 54 (2), 23–28.

[B59] Khattak S. Nguyen H. Mascarich F. Dang T. Alexis K. (2020). “Complementary Multi-Modal Sensor Fusion for Resilient Robot Pose Estimation in Subterranean Environments,” in 2020 International Conference on Unmanned Aircraft Systems (ICUAS) (Athens, Greece: Institute of Electrical and Electronics Engineers Inc.), 1024–1029. 10.1109/ICUAS48674.2020.9213865

[B60] Kim G. Yun S. Kim J. Kim A. (2022). “Sc-Lidar-Slam: A Front-End Agnostic Versatile Lidar Slam System,” in 2022 International Conference on Electronics, Information, and Communication (ICEIC), 1–6. 10.1109/iceic54506.2022.9748644

[B61] Klarer P. (1993). Space Robotics Programs at Sandia National Laboratories. Albuquerque, NM, USA: Sandia National Labs.

[B62] Komatsu (2023). Frontrunner Autonomous Haulage System (Ahs). Available online at: https://www.komatsu.com/en/site-optimization/smart-mining/loading-and-haulage/autonomous-haulage-system/.

[B63] Konieczna-Fulawka M. Koval A. Nikolakopoulos G. Fumagalli M. Santas Moreu L. Vigara-Puche V. (2025). Autonomous Mobile Inspection Robots in Deep Underground Mining-the Current State of the Art and Future Perspectives. Sensors (Basel) 25 (12), 3598. 10.3390/s25123598 40573485 PMC12196989

[B64] Kot T. Novák P. Babjak J. Olivka P. (2016). “Rendering of 3d Maps with Additional Information for Operator of a Coal Mine Mobile Robot,” in Modelling and Simulation for Autonomous Systems (Cham: Springer International Publishing).

[B65] Kot T. Bajak J. Novák P. (2017). “Analysis and Prevention of Selected Risks of Remotely and Autonomously Controlled Mobile Robot Telerescuer,” in 18th International Carpathian Control Conference (ICCC) (IEEE).

[B66] Koukis G. Safouri K. Tsaoussidis V. (2024). All About Delay-Tolerant Networking (Dtn) Contributions to Future Internet. Future Internet 16 (4), 129. 10.3390/fi16040129

[B67] Kowalski-Trakofler K. Alexander D. Brnich M. McWilliams L. Podlesny A. Lenart P. (2009). Underground Coal Mining Disasters and Fatalities--United States, 1900–2006. 19116608

[B68] Krotkov E. Bares J. Katragadda L. Simmons R. Whittaker R. (1994). “Lunar Rover Technology Demonstrations with Dante and Ratler,” in JPL, Third International Symposium on Artificial Intelligence, Robotics, and Automation for Space.

[B69] Lee M.-F. R. Chien T.-W. (2020). “Artificial Intelligence and Internet of Things for Robotic Disaster Response,” in 2020 International Conference on Advanced Robotics and Intelligent Systems (ARIS) (IEEE).

[B70] Li Y. T. Li M. G. Zhu H. Hu E. Y. Tang C. Q. Li P. (2020). Development and Applications of Rescue Robots for Explosion Accidents in Coal Mines. J. Field Robotics 37 (3), 466–489. 10.1002/rob.21920

[B71] Li M. Zhu H. Tang C. You S. Li Y. (2022). “Coal Mine Rescue Robots: Development, Applications and Lessons Learned,” in Proceedings of 2021 International Conference on Autonomous Unmanned Systems (ICAUS 2021) (Singapore: Springer Singapore).

[B72] Liang Y. Liu Y. Lu C. Cui D. Yang J. Zhou R. (2024). Research on Intelligent Monitoring and Protection Equipment of Vital Signs of Underground Personnel in Coal Mines: Review. Sensors (Basel) 25 (1), 63. 10.3390/s25010063 39796854 PMC11722757

[B73] Liu Q. Wang Y. Zhao F. Zheng C. Xie J. (2025a). A Review of the Research Progress of Sensor Monitoring Technology in Harsh Engineering Environments. Sensors (Basel) 25 (20), 6308. 10.3390/s25206308 41157362 PMC12567475

[B74] Liu H. Zhang Z. H. Dong J. Q. Benndorf J. Jia X. W. Wang X. F. (2025b). A Review of Positioning Technologies for Personnel and Equipment in Underground Mines. Int. J. Digital Earth 18 (1), 2506493. 10.1080/17538947.2025.2506493

[B75] Lööw J. (2020). “Soft' Questions in a 'Hard,” in Industry?

[B76] Lööw J. Nygren M. (2019). Initiatives for Increased Safety in the Swedish Mining Industry: Studying 30 Years of Improved Accident Rates. Saf. Sci. 117, 437–446. 10.1016/j.ssci.2019.04.043

[B77] Lopez P. Risso N. Anani A. Momayez M. (2024). Geohazard Identification in Underground Mines: A Mobile App. Sensors (Basel) 24 (24), 8052. 10.3390/s24248052 39771787 PMC11679759

[B78] Lopez-Arreguin A. J. R. Montenegro S. (2020). Towards Bio-Inspired Robots for Underground and Surface Exploration in Planetary Environments: An Overview and Novel Developments Inspired in Sand-Swimmers. Heliyon 6 (6), e04148. 10.1016/j.heliyon.2020.e04148 32613101 PMC7317692

[B79] Luo B. Will-Cole A. R. Dong C. Z. He Y. F. Liu X. X. Lin H. D. (2024). Magnetoelectric Microelectromechanical and Nanoelectromechanical Systems for the Iot. Nat. Rev. Electr. Eng. 1 (5), 317–334. 10.1038/s44287-024-00044-7

[B80] Ma H. Liu E. Ni W. Fang Z. Wang R. Gao Y. (2025). Through-the-Earth Magnetic Induction Communication and Networking: A Comprehensive Survey. IEEE Commun. Surv. and Tutorials. 10.1109/COMST.2025.3623258

[B81] Maity A. Majumder S. Ray D. N. (2013). “Amphibian Subterranean Robot for Mine Exploration,” in 2013 International Conference on Robotics, Biomimetics, Intelligent Computational Systems. 10.1109/ROBIONETICS.2013.6743612

[B82] Mardonova M. Choi Y. (2018). Review of Wearable Device Technology and Its Applications to the Mining Industry. Energies 11 (3), 547. 10.3390/en11030547

[B83] Martz J. Al-Sabban W. Smith R. N. (2020). Survey of Unmanned Subterranean Exploration, Navigation, and Localisation. Iet Cyber-Systems Robotics 2 (1), 1–13. 10.1049/iet-csr.2019.0043

[B6] Masayuki A. Takayama T. Hirose S. (2004). “Development of “Souryu-III”: connected crawler vehicle for inspecfion inside narrow and winding spaces,” in 2004 IEEE/RSJ International Conference on Intelligent Robots and Systems (IROS). (Japan: EEE Cat. No.04CH37566), 52–57. 10.1109/iros.2004.1389328

[B84] McAteer J. D. (2006). The Sago Mine Disaster a Preliminary Report to Governor Joe Manchin Iii.

[B85] McDonald P. (2023). *Hopes Fading for Trapped Miners*. 2010 07/06/2023. Available online at: https://www.abc.net.au/news/2010-11-23/hopes-fading-for-trapped-miners/2347394 (Accessed July 6, 2023).

[B86] Moczulski W. Cyran K. Novak P. Rodriguez A. Januszka M. (2014). “Telerescuer-a Concept of a System for Teleimmersion of a Rescuer to Areas of Coal Mines Affected by Catastrophes,” in VI Międzynarodowa Konferencja Systemy Mechatroniczne Pojazdów i Maszyn Roboczych.

[B87] Molyneaux L. Carnegie D. A. Hades C. C. (2015). “An Underground Mine Disaster Scouting Robot,” in 2015 IEEE International Symposium on Safety, Security, and Rescue Robotics (SSRR) (IEEE).

[B88] Morris A. Ferguson D. Omohundro Z. Bradley D. Silver D. Baker C. (2006). Recent Developments in Subterranean Robotics. J. Field Robotics 23 (1), 35–57. 10.1002/rob.20106

[B89] MSHA and NMSHA, *Advanced Mine Rescue Training (Coal Mines)* (2013). Mine Safety and Health Administration, National Mine Health and Safety Academy

[B90] MSHA (2006). in Report of Investigation: Fatal Underground Coal Mine Explosion. Editors Light T. E. Herndon R. C. Guley, Jr. P. E. A. R. Cook, Sr. G. L. Odum M. A. Bates, JR. R. M. (Arlington, VA: U.S. Department of Labor, Mine Safety and Health Administration).Sago Mine, Wolf Run Mining Company, Tallmansville, Upshur County, West Virginia

[B91] MSHA (2007). in Internal Review of MSHA’s Actions at the Sago Mine Wolf Run Mining Company, Sago, Upshur County, West Virginia. Editors Chao E. L. Stickler R. E. (Arlington, VA: U.S. Department of Labor, Mine Safety and Health Administration).

[B92] Murphy R. R. (2010). Navigational and Mission Usability in Rescue Robots. J. Robotics Soc. Jpn. 28 (2), 142–146. 10.7210/jrsj.28.142

[B93] Murphy R. R. (2014). Disaster Robotics. MIT press.

[B94] Murphy R. R. Kravitz J. Stover S. L. Shoureshi R. (2009). Mobile Robots in Mine Rescue and Recovery. Ieee Robotics and Automation Mag. 16 (2), 91–103. 10.1109/Mra.2009.932521

[B95] Muslim H. Itoh M. (2019). A Theoretical Framework for Designing Human-Centered Automotive Automation Systems. Cognition Technol. and Work 21 (4), 685–697. 10.1007/s10111-018-0509-8

[B96] NASA (2019). Nasa's Centennial Challenges: Space Robotics Challenge. Available online at: https://www.nasa.gov/prizes-challenges-and-crowdsourcing/centennial-challenges/past-challenges/ (Accessed July 13, 2026).

[B97] NIOSH (2023). Advancing Self-Escape Training: A Needs Analysis Based on the National Academy of Sciences Report, 'Improving Self-Escape from Underground Coal Mines'. By Hoebbel, Cl, Bellanca, Jl, Ryan, Me, Brnich, Mj. Pittsburgh PA: U.S. Department of Health and Human Services, Centers for Disease Control and Prevention, National Institute for Occupational Safety and Health. 10.26616/NIOSHPUB2023134

[B98] Novák P. Kot T. Babjak J. Konečný Z. Moczulski W. Rodriguez López Á. (2018). Implementation of Explosion Safety Regulations in Design of a Mobile Robot for Coal Mines. Appl. Sci. 8 (11), 2300. 10.3390/app8112300

[B99] Occupational Safety and Health Administration (1998). OSHA Information Booklet #3143 -- Industrial Hygiene. Available online at: https://www.osha.gov/publications/OSHA3143#What%20Are (Accessed April 30, 2023).

[B100] Okamura A. M. Mataric M. J. Christensen H. I. (2010). Medical and Health-Care Robotics. Ieee Robotics and Automation Mag. 17 (3), 26–37. 10.1109/Mra.2010.937861

[B101] Opiyo S. Zhou J. Mwangi E. Kai W. Sunusi I. (2021). A Review on Teleoperation of Mobile Ground Robots: Architecture and Situation Awareness. Int. J. Control, Automation Syst. 19, 1384–1407. 10.1007/s12555-019-0999-z

[B102] Papachristos C. Khattak S. Mascarich F. Alexis K. (2019). “Autonomous Navigation and Mapping in Underground Mines Using Aerial Robots,” in 2019 IEEE Aerospace Conference (IEEE).

[B103] Parasuraman R. Sheridan T. B. Wickens C. D. New Collective A. (2000). A Model for Types and Levels of Human Interaction with Automation. IEEE Trans. Syst. Man. Cybern. A Syst. Hum. 30 (3), 286–297. 10.1109/3468.844354 11760769

[B104] Paredes D. Fleming-Muñoz D. (2021). “Automation and Robotics in Mining: Jobs, Income and Inequality Implications,” in Extractive Industries and Society (Elsevier Ltd.), 189–193. 10.1016/j.exis.2021.01.004

[B105] Petrlik M. Petracek P. Kratky V. Musil T. Stasinchuk Y. Vrba M. (2022). Uavs beneath the Surface: Cooperative Autonomy for Subterranean Search and Rescue in Darpa Subt.arXiv Preprint arXiv:2206.08185. 10.48550/arXiv.2206.08185

[B106] Pratt G. Manzo J. (2013). “The Darpa Robotics Challenge,” in IEEE Robotics and Automation Magazine, 10–12. 10.1109/MRA.2013.2255424

[B107] Purvis J. W. Klarer P. R. (1994). Robotic All-Terrain Lunar Exploration Rover.

[B108] Queralta J. P. Taipalmaa J. Pullinen B. C. Sarker V. K. Gia T. N. Tenhunen H. (2020). Collaborative Multi-Robot Systems for Search and Rescue: Coordination and Perception. arXiv Preprint arXiv:2008.12610. 10.48550/arXiv.2008.12610

[B109] Ralston J. C. Hainsworth D. W. (1998). “The Numbat: A Remotely Controlled Mine Emergency Response Vehicle,” in Field and Service Robotics (Springer).

[B110] Ralston J. C. Hainsworth D. W. Reid D. C. Anderson D. L. McPhee R. J. (2001). Recent Advances in Remote Coal Mining Machine Sensing, Guidance, and Teleoperation. Robotica 19 (5), 513–526. 10.1017/S0263574701003447

[B111] Ralston J. Reid D. Hargrave C. Hainsworth D. (2014). Sensing for Advancing Mining Automation Capability: A Review of Underground Automation Technology Development. Int. J. Min. Sci. Technol. 24 (3), 305–310. 10.1016/j.ijmst.2014.03.003

[B112] Ranjan A. Sahu H. B. Misra P. (2020). Modeling and Measurements for Wireless Communication Networks in Underground Mine Environments. Measurement 149, 106980. 10.1016/j.measurement.2019.106980

[B113] Ray D. N. Dalui R. Maity A. Majumder S. (2009). Sub-Terranean Robot: A Challenge for the Indian Coal Mines. Online J. Electron. Electr. Eng. (OJEEE) 2 (2), 217–222.

[B114] Reddy A. H. Kalyan B. Murthy C. S. N. (2015). Mine Rescue Robot System – a Review. Procedia Earth Planet. Sci. 11, 457–462. 10.1016/j.proeps.2015.06.045

[B115] Reuters (2010). “Robots Move up Nz Coal Mine,” in Survival Hopes Fade.

[B116] RoboCup Federation (2023). Robocuprescue. Available online at: https://www.robocup.org/domains/2 (Accessed August 14, 2023).

[B117] Rong X. Song R. Song X. Li Y. (2011). “Mechanism and Explosion-Proof Design for a Coal Mine Detection Robot,” in Procedia Engineering. 10.1016/j.proeng.2011.08.021

[B118] Roucek T. Pecka M. Cızek P. Petrıcek T. Bayer J. Šalansky V. (2021). System for Multi-Robotic Exploration of Underground Environments Ctu-Cras-Norlab in the Darpa Subterranean Challenge.arXiv Preprint arXiv:2110.05911. 10.48550/arXiv.2110.05911

[B119] RT News (2016). Robots Deployed in Search Operation Following Deadly Russian Coal Mine Explosion (Video). Available online at: https://www.rt.com/news/334038-robots-vorkuta-coal-mine/ (Accessed June 28, 2023).

[B120] Ruckelshausen A. Biber P. Dorna M. Gremmes H. Klose R. Linz A. (2009). Bonirob: An Autonomous Field Robot Platform for Individual Plant Phenotyping. Precision Agriculture. 10.1163/9789086866649_101

[B121] Sadeghi S. Soltanmohammadlou N. Nasirzadeh F. (2022). Applications of Wireless Sensor Networks to Improve Occupational Safety and Health in Underground Mines. J. Saf. Res. 83, 8–25. 10.1016/j.jsr.2022.07.016 36481040

[B122] Salonitis K. (2014). Modular Design for Increasing Assembly Automation. Cirp Annals-Manufacturing Technol. 63 (1), 189–192. 10.1016/j.cirp.2014.03.100

[B123] Salton J. R. Hobart C. G. Garretson J. Callow D. S. (2017). *Mine Rescue Robotics: Gemini Scout-18507*. 2017. Albuquerque, NM (United States): Sandia National Lab.SNL-NM.

[B124] Sammarco J. Wesh J. Reyes M. Ruff T. Sunderman C. (2018). “Mine of the Future: Disruptive Technologies That Impact Our Future Mine Worker Health and Safety Research Focus,” in 2018, Internal NIOSH report, 57–62.

[B125] Sapaty P. (2015). Military Robotics: Latest Trends and Spatial Grasp Solutions. Int. J. Adv. Res. Artif. Intell. 4 (4), 9–18. 10.14569/IJARAI.2015.040402

[B126] Saxena P. Shukla P. (2023). A Review on Recent Developments and Advances in Environmental Gas Sensors to Monitor Toxic Gas Pollutants. Environ. Prog. and Sustain. Energy 42 (5), e14126. 10.1002/ep.14126

[B127] Sebastian A. (2025). Soft Robotics for Search and Rescue: Advancements, Challenges, and Future Directions. arXiv Preprint arXiv:2502.12373. 10.48550/arXiv.2502.12373

[B128] Semeraro F. Griffiths A. Cangelosi A. (2022). “Human–Robot Collaboration and Machine Learning: A Systematic Review of Recent Research,” in Robotics and Computer-Integrated Manufacturing (Elsevier Ltd.). 10.1016/j.rcim.2022.102432

[B129] Shahmoradi J. Talebi E. Roghanchi P. Hassanalian M. (2020). A Comprehensive Review of Applications of Drone Technology in the Mining Industry. Drones 4 (3), 34. 10.3390/drones4030034

[B130] Sheridan T. Verplank W. (1978). Human and Computer Control of Undersea Teleoperators (Tech. Rep.). Man-Machine Systems Laboratory. Cambridge, MA: Massachusetts Institute of Technology.

[B131] Singh N. Gunjan V. K. Chaudhary G. Kaluri R. Victor N. Lakshmanna K. (2022). Iot Enabled Helmet to Safeguard the Health of Mine Workers. Comput. Commun. 193, 1–9. 10.1016/j.comcom.2022.06.032

[B132] Svertoka E. Saafi S. Rusu-Casandra A. Burget R. Marghescu I. Hosek J. (2021). Wearables for Industrial Work Safety: A Survey. Sensors (Basel) 21 (11), 3844. 10.3390/s21113844 34199446 PMC8199604

[B133] Szczurek K. A. Prades R. M. Matheson E. Rodriguez-Nogueira J. Castro M. D. (2023). Multimodal Multi-User Mixed Reality Human-Robot Interface for Remote Operations in Hazardous Environments. Ieee Access 11, 17305–17333. 10.1109/Access.2023.3245833

[B134] Szrek J. Zimroz R. Wodecki J. Michalak A. Góralczyk M. Worsa-Kozak M. (2020). Application of the Infrared Thermography and Unmanned Ground Vehicle for Rescue Action Support in Underground Mine—the Amicos Project. Remote Sens. 13 (1), 69. 10.3390/rs13010069

[B135] Thrun S. Thayer S. Whittaker W. Baker C. Burgard W. Ferguson D. (2004). Autonomous Exploration and Mapping of Abandoned Mines - Software Architecture of an Autonomous Robotic System. Ieee Robotics and Automation Mag. 11 (4), 79–91. 10.1109/Mra.2004.1371614

[B136] Tranzatto M. Miki T. Dharmadhikari M. Bernreiter L. Kulkarni M. Mascarich F. (2022). Cerberus in the Darpa Subterranean Challenge. Sci. Robot. 7 (66), eabp9742. 10.1126/scirobotics.abp9742 35613301

[B137] Wang W. D. Dong W. Su Y. Y. Wu D. M. Du Z. J. (2014). Development of Search-and-Rescue Robots for Underground Coal Mine Applications. J. Field Robotics 31 (3), 386–407. 10.1002/rob.21501

[B138] Wang Y. Tian P. Zhou Y. Chen Q. (2018). The Encountered Problems and Solutions in the Development of Coal Mine Rescue Robot. J. Robotics 2018 (1), 8471503–11. 10.1155/2018/8471503

[B139] Wang Q. Lyu P. Lai J. Z. Bai S. Y. Fang W. Li J. (2025). Research Progress on Collaborative Perception and Localization Technologies of Clustered Unmanned Systems in Underground Sheltered Spaces: A Survey. Ieee Trans. Instrum. Meas. 74, 1–27. 10.1109/Tim.2025.3600832 42146727

[B140] Whitman J. Zevallos N. Travers M. Choset H. (2017). Snake Robot Urban Search after the 2017 Mexico City Earthquake.

[B141] Zhai G. D. Zhang W. T. Hu W. Y. Ji Z. D. (2020). Coal Mine Rescue Robots Based on Binocular Vision: A Review of the State of the Art. Ieee Access 8, 130561–130575. 10.1109/Access.2020.3009387

[B142] Zhao J. Gao J. Zhao F. Liu Y. (2017). A Search-and-Rescue Robot System for Remotely Sensing the Underground Coal Mine Environment. Sensors (Basel) 17 (10). 10.3390/s17102426 29065560 PMC5677175

[B143] Zhao J. Han T. Wang S. Liu C. Fang J. Liu S. (2021). Design and Research of All-Terrain Wheel-Legged Robot. Sensors (Basel) 21 (16), 5367. 10.3390/s21165367 34450814 PMC8401446

[B144] Zou M. Zhu J. Z. Wang K. Lin Y. C. Jin J. F. He L. B. (2020). Design and Mechanical Behavior Evaluation of Flexible Metal Wheel for Crewed Lunar Rover. Acta Astronaut. 176, 69–76. 10.1016/j.actaastro.2020.06.010

