# Supplementary material for: Robots in mine search and rescue operations: a review of platforms and design requirements
Source: Front Robot AI. 2026 Aug 5;13:1857704. doi: 10.3389/frobt.2026.1857704 (PMC13487453; doi:10.3389/frobt.2026.1857704)
Supplement: Supplementary file 1 [file Supplementaryfile1.docx]

Appendix A: IEC 60079 Standards Series List

Table A.1: Overview of IEC 60079 Standards (obtained from descriptions at https://webstore.iec.ch/publication/32878)

| **IEC Standard - Part** | **Description** |
| --- | --- |
| IEC 60079-0:2017 | **General requirements** for construction, testing and marking of Ex Equipment and Ex Components intended for use in explosive atmospheres. |
| IEC 60079-1:2014 | Equipment protection by **flameproof enclosures** - protection type "d" - for explosive gas atmospheres |
| IEC 60079-2:2014 | Equipment protection by **pressurized enclosures** - protection type "p" - for explosive gas atmospheres or explosive dust atmospheres, and enclosures containing a limited release of a flammable substance. |
| IEC 60079-5:2015 | Equipment protection by **powder filling -** protection type "q" - for explosive gas atmospheres |
| IEC 60079-6:2015 | Equipment protection by **liquid immersion -** protection type "o" - for explosive gas atmospheres |
| IEC 60079-7:2015 | Equipment protection by **increased safety** - protection type "e" - for explosive gas atmospheres. Applies to equipment or Ex Components, including their connections, conductors, windings, lamps, and batteries; and also including semiconductors and electrolytic capacitors |
| IEC 60079-10-1:2020 | **Classification of areas** - Explosive gas atmospheres (with flammable gas or vapour hazards) |
| IEC 60079-10-2:2015 | **Classification of areas** - Explosive dust atmospheres and combustible dust layers |
| IEC 60079-11:2023 | Equipment protection by **intrinsic safety** - protection type "i" - for explosive atmospheres |
| IEC 60079-13:2017 | Equipment protection by **pressurized room** "p" and **artificially ventilated room** "v" |
| IEC 60079-14:2013 | Electrical installations design, selection, erection and initial inspection of electrical installations in, or associated with, explosive atmospheres |
| IEC 60079-15:2017 | Equipment protection by **sealing, hermetical sealing, non-incentive components and restricted breathing enclosures** - protection type "n" - for explosive gas atmospheres |
| IEC 60079-16:1990 | Artificial ventilation for the protection of analyser(s) houses |
| IEC 60079-17:2013 | Electrical installations inspection and maintenance within hazardous areas (flammable gases, vapours, mists, dusts, fibres or flyings) |
| IEC 60079-18:2014 | Electrical Equipment protection **by encapsulation -** protection type "m" - for explosive gas atmospheres or explosive dust atmospheres |
| IEC 60079-19:2019 | Equipment repair, overhaul, reclamation and modification of equipment designed for use in explosive atmospheres |
| IEC 60079-25:2020 | Design, construction and assessment of intrinsically safe systems -protection type “i” - intended for use, as a whole or in part, in locations in which the use of Group I, II or III Ex Equipment is required |
| IEC 60079-26:2021 | Equipment with different Equipment Protection Levels (EPLs) and a separation element |
| IEC 60079-28:2015 | Protection of equipment and transmission systems emitting optical radiation for explosive atmospheres |
| IEC 60079-29-1:2016 | Gas detectors - Performance requirements of detectors for flammable gases or vapour concentrations with air |
| IEC 60079-29-2:2015 | Gas detectors - Selection, installation, use and maintenance of detectors for flammable gases and oxygen |
| IEC 60079-29-3:2014 | Gas detectors - Guidance on functional safety of fixed gas detection systems |
| IEC 60079-29-4:2009 | Gas detectors - Performance requirements of open path detectors for flammable gases |
| IEC 60079-31:2022 | Equipment dust ignition protection by enclosure and surface temperature limitation - protection type "t" - for explosive dust atmospheres |
| IEC TS 60079-32-1:2013 | Electrostatic hazards - Guidance |
| IEC TS 60079-32-2:2015 | Electrostatics hazards - Tests |
| IEC 60079-33:2012 | Equipment protection by special protection - protection type "s" (protection not covered by any existing standard in the IEC 60079 series) |
| IEC 60079-35-1:2011 | Caplights for use in mines susceptible to firedamp - General requirements - Construction and testing in relation to the risk of explosion |
| IEC 60079-35-2:2011 | Caplights for use in mines susceptible to firedamp - Performance and other safety-related matters |
| IEC TS 60079-39:2015 | Intrinsically safe systems with electronically controlled spark duration limitation |
| IEC TS 60079-40:2016 | Requirements for process sealing between flammable process fluids and electrical systems |
| IEC TS 60079-42:2019 | Electrical safety devices for the control of potential ignition sources for Ex-Equipment |
| IEC TS 60079-43:2017 | Equipment in adverse service conditions in explosive atmospheres (e.g., ambient temperatures below -20 °С, including maritime applications) |
| IEC TS 60079-46:2017 | Equipment assemblies |
| IEC TS 60079-47:2021 | Equipment protection by 2-wire intrinsically safe ethernet concept (2-WISE) |
